# Application of DIC Method in the Analysis of Stress Concentration and Plastic Zone Development Problems

**DOI:** 10.3390/ma13163460

**Published:** 2020-08-05

**Authors:** Paweł J. Romanowicz, Bogdan Szybiński, Mateusz Wygoda

**Affiliations:** 1 Institute of Machine Design, Cracow University of Technology, ul. Warszawska 24, 31-155 Cracow, Poland; pawel.romanowicz@pk.edu.pl; 2 Department of Product Technology and Ecology, College of Management and Quality Sciences, Cracow University of Economics, ul. Rakowicka 27, 31-510 Cracow, Poland; mateusz.wygoda@uek.krakow.pl

**Keywords:** digital image correlation, stress concentration factor, notches, static tension tests, finite element analysis, microstructure analysis

## Abstract

The paper presents the assessment of the possibility and reliability of the digital image correlation (DIC) system for engineering and scientific purposes. The studies were performed with the use of samples made of the three different materials—mild S235JR + N steel, microalloyed fine-grain S355MC steel, and high strength 41Cr4 steel subjected to different heat-treatment. The DIC studies were focused on determinations of dangerous zones with large stress concentrations, plastic deformation growth, and prediction of the failure zone. Experimental tests were carried out for samples with different notches (circular, square, and triangular openings). With the use of the DIC system and microstructure analyses, the influence of different factors (laser cutting, heat treatment, material type, notch shape, and manufacturing quality) on the material behavior were studied. For all studied cases, the stress concentration factors (SCF) were estimated with the use of the analytical formulation and the finite element analysis. It was observed that the theoretical models for calculations of the influence of the typical notches may result in not proper values of SCFs. Finally, the selected results of the total strain distributions were compared with FEM results, and good agreement was observed. All these allow the authors to conclude that the application of DIC with a common digital camera can be effectively applied for the analysis of the evolution of plastic zones and the damage detection for mild high-strength steels, as well as those normalized and quenched and tempered at higher temperatures.

## 1. Introduction

Emerging methods of manufacturing, new technologies in materials production [1,2,3,4,5], and new modeling techniques result in more and more complex machines, subassemblies, and shapes of structural elements in use [6,7,8,9,10,11,12,13,14]. Both the designed structures and the materials used for its manufacturing should fulfill strict standards and expected demands to provide safe operation. This can be observed in the area of pressure vessel design, bolted connections, etc. These observations become a challenge for contemporary designers and machine constructors, making the designing process complex and knowledge demanding [6,15]. On the other hand, machine or structure design for several years has become a compromise between the cost and time spent on designing. The contemporary designed structural machine elements are increasingly complex and may include certain shape modifications, e.g., holes or openings [16,17,18,19,20,21,22,23,24,25]; grooves, shoulders, and undercuts [26,27,28,29,30,31]; threads [10]; keyways [9]; welded connections [13,32]; etc. Such disturbance of the geometry disrupts the flow of the internal forces and causes the local high-stress occurrence, which is sometimes several times higher than the nominal stress calculated with simple basic formulas [33,34,35]. The raise of the stress level is evaluated by the stress concentration factor (SCF), which has been introduced for typical shape and geometry changes or discontinuities [14,17,19]. Additionally, safety requirements, compliance with codes, and environmental standards make the designing process a real challenge in the case of new or complex machines. Based on this, two general approaches for machine and structure designing have been adopted, namely fail-safe design and safe life design, and are successfully used in practice [14,15]. However, still unexpected, premature failures of machines may sometimes appear and every designer must be aware that it is not possible to provide full reliability and safety of the designed object [14,36,37].

In the area of the material production still, new materials are issued, starting from very modern composite materials and ending with the new steel alloys [1,2,3,4,5,38,39]. The introduction of new materials is accompanied by the development of the more and more complex material models [40] and fatigue criteria [41,42], which better and better describe the material and structure response under the applied load systems [42]. These models are effectively used in numerical simulations, which broaden the knowledge about the possible structure behavior, particularly under extreme conditions. Such a situation appears in the case of structural elements made from steel in the regime of large plastic deformations or in elements with stress raisers such as cracks, voids, faults, etc. [43]. Searching for the growth and spread of plastic zones in ductile materials and the crack nucleation and evolution in brittle materials give the researchers the knowledge about the possible failure mechanisms, which were not available until recently [44]. The theoretical analyses and numerical simulations are supplemented by extensive experimental investigations. In this area, the non-destructive and non-contact methods have become the essential tool of the contemporary experimental analysis [45].

Recently, several non-destructive approaches for failure analysis and damage detection have been developed at the turn of the XX–XXI century. These are techniques based on acoustic emission, lamb wave propagation, application of thermography cameras, the Moire interferometry, or scanning electron microscopy [46]. In the 1980s, the non-destructive and contactless optical method called the digital image correlation (DIC) method was set [47,48,49]. Such a technique, also called electronic speckle photography, computer-aided speckle interferometry, texture correlation, or speckle correlation method, was based on the image acquisition of the inspected surface before and after loading and calculation of surface deformation in post-processing analysis with the use of correlation criteria. In the beginning, the application of the DIC method was strongly limited by the computer speed and the size of the files to process. The rapid development of the computing power and storage possibilities enabled that method to become the leading one in the non-destructive analysis of the structure deformations [50]. Such investigation is done with the use of the high-resolution digital cameras and the respective correlation function [51]. The application of the binocular stereovision [45] allows for the inspection of objects with curved surfaces. The main issue of the DIC functioning is tracking and calculations of the motion of points on the investigated surface. For this purpose, the images of the structure with speckle patterns are split up for a virtual grid of subsets containing some number of points. The calculations and matching of the particular subsets (translation and deformation) are made with the use of correlation criteria. Based on the deformation map of the particular subsets, the distribution of displacements and strains can be produced. The detailed description of the different algorithms applied in DIC software and the basics of the DIC technique can be found in [51]. Generally, the DIC is used for detection and characterization of the surface damage [50,52,53,54,55,56,57], performance analyses of the structural objects [58,59,60,61], characterization of material properties [62,63], and damage detection under impact loadings [64,65,66]. The precision of the method depends on the camera resolution, quality of its optics, light intensity, and others. With the use of the professional DIC system, it is possible to achieve good quantitative and qualitative calculation of displacements and strains fields on the investigated surface nowadays. However, the method has one important disadvantage, which may be limiting its application, i.e., the size of the images, which can be very large and sometimes difficult in the computer process.

The above-mentioned problem of stress concentration analysis has attracted researchers for many years. The primary mathematical solutions for the notch problem within the elastic range was set for the infinite flat and thin infinite element with a circular hole and published by Kirsch [33], Kolosov [34], and Inglis [67]. The fundamental analytical solutions for displacement, strain, and stress distributions in elastic isotropic and anisotropic structural elements with notches were later published in works of Lekhnitskiy [68], Mushkelishvili [69], Neuber [35], Savin [17], and others [14,18,70,71,72,73]. The elaborated formulations mainly concerned the unbounded plates with openings or other stress raisers subjected to simple or complex systems of loads. The results for openings of circular, elliptical, rectangular, or triangular shapes were of the primary interest, and the influence of their spatial orientations on the results was also studied [16,22,68]. These pure mathematical formulas and solutions concerning the stress concentration assessment are usually complex and their applications are rather limited [19,23]. The above-mentioned solutions usually give unreal values for the real structural elements with finite size and dimensions and the suitable finite width correction factors should be introduced in practical use [16]. The assessment of the size and dimension influence on the stress concentration was mainly possible due to the development of the experimental methods [18,74,75]. These were pushed forward by the construction of the contemporary polariscopes utilizing the effect of forced birefringence. Such a phenomenon appears in certain transparent isotropic materials when unloaded, if these are placed under the load system certain fringe patterns can be observed. Based on them, after certain manipulations, the stress distribution can be assessed. This method was of particular meaning for plane stress problems and dozens of charts and plots have been worked out for numerous stress raisers. The intensive progress in this area was summarized in several books and recommendations for mechanical, structural, transport, rail, civil, or bridge engineering [14,19,76]. One of the most popular recommendations for the designing process is the Engineering Sciences Data Unit (ESDU—homepage with software and formulas for designing) [77], which brings proved and validated methods for designing and covers a wide range of disciplines. The common alternative for the above-given approaches became the numerical methods [21,23]. In this area, the Finite Element Method (FEM) became the leading one. Nowadays, a many effective computer commercial and scientific FEM software exist, which are the basic tool for scientific and designing applications. Additionally, the complex material models [40] can be implemented and large strain effects, the presence, and growth of plastic zones can be analyzed more effectively.

The main aim of this paper is the assessment of possibilities and reliability of the DIC system for the determinations of dangerous zones with large stress concentration, plastic deformation growth, and prediction of the failure zone and mode for different structural steels. For this reason, DIC analyses were performed for different steels with different notches and some of them were compared with the results obtained with the use of the classical FEM analysis. The other aims of the paper are as follows:

(1) Verification of the theoretical models for typical openings by the experiments and FEM simulations; and
(2) Evaluation of the influence of the different notches (square, triangular, and circular), manufacturing technologies (laser cutting), material type (including relatively new material microalloyed fine-grain hot rolled S355MC steel), and microstructure (softened, hardened) on the structure and material behavior, plastic deformation growth, and failure mode.

The paper consists of eight sections. The introduction to the investigated problems and the literature review is described in Section 1. The description of the tested materials and methods and specimens used in the analyses is presented in Section 2 and Section 3. The results of the theoretical and numerical studies of the SCF for notches used in the study as well as information about notch sensitivities of the used materials are given and discussed in Section 4. The results of the experimental tensile tests are presented in Section 5. The results of the DIC analyses and comparison of the DIC results with FEM are illustrated and discussed in Section 6. The obtained results are supplemented by the microstructure analysis given in Section 7. The final discussion taking into account all methods applied during as well as after experimental tests are given in Section 8. The conclusions are given in Section 9.

## 2. Materials and Methods

To investigate the influence of the notch effect in structural elements as well as the possibilities of prediction of damage position and form, the three different materials (S235JR + N, S355MC, and 41Cr4—see Table 1 and Table 2) have been tested in the present study. The selected structural steels are commonly used for the manufacturing of the mechanical parts and structures. Non-alloy structural steel S235JR exhibits good weldability and mechanical properties and is commonly used for manufacturing of welded structures, typical mechanical parts, and beams, spans of bridges, columns, and structural elements in civil engineering. Microalloyed, low carbon, and fine-grain S355MC structural steel can be classified into high-strength, low-alloy steels, which also exhibits high ductility. It also has good weldability and additionally offers good fatigue properties. In the final production procedure, the steel is usually subjected to thermo-mechanical rolling. This type of material is commonly used in the automobile industry due to its extended cold forming and bending possibilities. The steel 41Cr4 is the material which poses its high-level strength properties after quenching and tempering and is used for components such as gear wheels, axles, crankshafts, levers, etc., where high-temperature resistance, good abrasive properties, and other high mechanical properties are demanded. When this steel is in the annealed condition, it is easily machined and cut. The 41Cr4 steel is particularly predicted for induction surface hardening, which significantly improves its mechanical properties. However, after hardening its notch sensitivity factor becomes close to 1.0. The fatigue properties of 41Cr4 strongly depend on the final treatment.

The investigated steel S235JR + N was produced in basic oxygen furnace (Linz-Donawitz process) steelmaking process and hot-rolled with coils thickness 2 mm and tested after normalizing rolling (+N). The influence of normalization on the mechanical properties was discussed by Rdzawski et al. [78]. Steel S355MC was produced in hot-rolled plain sheets. Steel 41Cr4 was cold-rolled and tested after hardening (41Cr4 + Q; hardened in the temperature 860 °C and quenched in oil), heat treatment (41Cr4 + QT; hardened in the temperature 860 °C, quenched in oil, tempered at 600 °C, and cooled down to the room temperature with an oven), and spheroidizing annealing (41Cr4 + AC). The hardnesses of the investigated samples were measured after heat treatment and were equal to 54HRC for 41Cr4 + Q and 35HRC for 41Cr4 + QT.

The investigated materials were certificated with respect to the recent standards [79,80,81]. The chemical composition and the mechanical properties of the particular materials are given in Table 1 and Table 2.

## 3. Specimens

**Figure 1:**
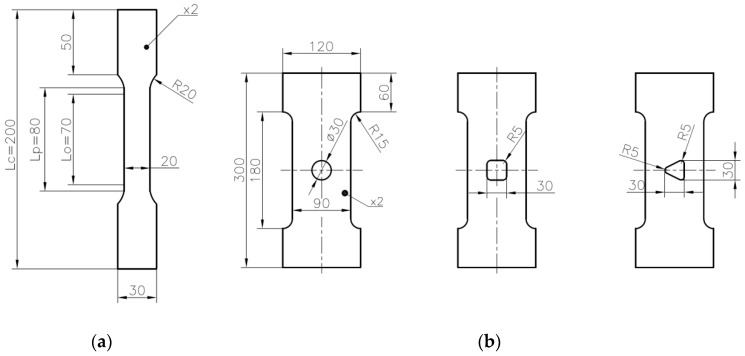
Dimension of samples made of S235JR + N steel: (**a**) Samples 1–3; and (**b**) Samples 4–6.

**Figure 2:**
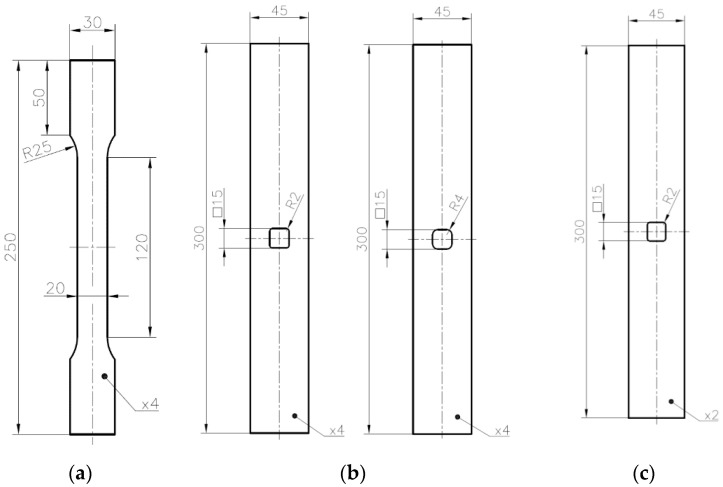
Dimension of investigated samples: (**a**) S355MC, Samples 7–8; (**b**) S355MC, Samples 9–10; and (**c**) 41Cr4, Samples 11–15.

The experimental tensile tests were performed for flat samples with different geometries and the three different materials mentioned above. The mechanical properties of the tested materials are described in Section 2. First, the static tensile tests at the room temperature were performed for S235JR + N and S355MC steels. The detailed information about the performed tests, including the loading rate, are given in Table 3. The geometries of the investigated samples followed the Standards [82] and are given in Figure 1a for steel S235JR + N and in Figure 2a for steel S355MC. These tests were performed for the determination of the tensile curves for further finite element numerical analyses purposes. In the next step, the tensile tests were performed for samples with different notches in the form of holes and made of different materials. The geometries of all tested samples are given in Figure 1b and Figure 2b,c. The wider samples with the width of 90 mm (Figure 1b) were made of S235JR + N and three different notches were, respectively, cut—a circular one with diameter φ30 mm, a square one 30 mm × 30 mm with corner fillet radii 5 mm, and a triangular (equilateral triangle) one with vertex fillet radii 5 mm and its span length equal to 30 mm. In all investigated specimens, the notches were located in the centers of the samples. The common feature of all investigated S235JR + N samples was the same size of the net cross-section area and the same technology—the high-pressure water jet cutting—used for their preparation. All these samples were used for the determination of the influence of the stress concentrator shape on the plastic zone development.

The samples made of S355MC had rectangular shape (Figure 2b) with square notch 15 mm × 15 mm with the side length set to 15 mm and rounded corners applied. Two samples with different corner radius (2 and 4 mm) were investigated. In this case, the main aim of the test was to study the influence of fillet radii in the square hole on the strain distributions. The samples made of 41Cr4 steel had the same rectangular shape and the notch in the form of the square hole but only one value of the corner radius was used. In the case of the 41Cr4 alloy steel, different heat treatments can be applied to improve its mechanical properties. Three samples made from 41Cr4 alloy steel were tested to study the influence of microstructure on the sensitivity of the material for stress concentrations and possibilities of the DIC application. These samples were subjected to different heat treatments. The first sample was normalized, the second one was quenched and tempered, and the third one was only quenched. The opening in the samples made of S355MC and 41Cr4 were cut by a laser.

The thicknesses of the tested samples were set to 2 mm for S235JR + N and 41Cr4 and 4 mm for S355MC; more details are given in Figure 1 and Figure 2 and Table 3.

**Figure 3:**
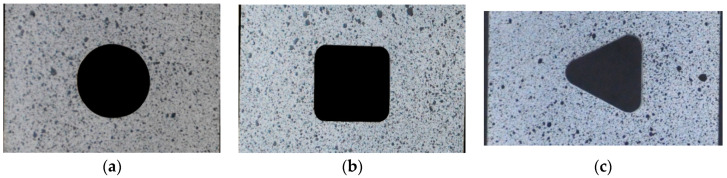
Mid-side parts of samples with holes made of S235JR + N and coated by speckle pattern: (**a**) circular hole φ30; (**b**) rectangular hole; and (**c**) triangular hole.

**Figure 4:**
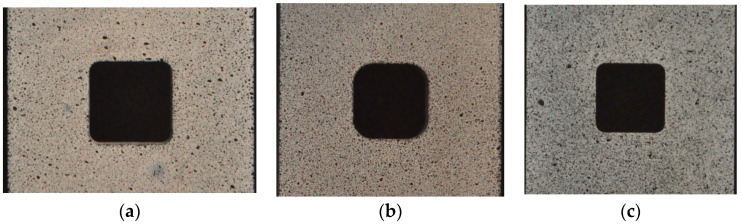
Mid-side parts of samples with rectangular holes coated by speckle pattern: (**a**) S355MC, radius 2 mm; (**b**) S355MC, radius 4 mm; and (**c**) 41Cr4 radius 2 mm.

To get the experimental distribution of the displacements and strains using the DIC analysis, the surfaces of all investigated samples were coated by randomly distributed speckle patterns with various unique shapes (Figure 3 and Figure 4). The first the white paintings of the investigated surfaces were made, after which the black aerosol speckles were sprayed on them. The photographs of undeformed and deformed samples under external tension loadings were made with the use of the Nikon D90 camera with Nikon AF-S NIKKOR 50 mm f/1.8 G lens. The camera was additionally equipped with the shutter release, which provided the shooting of photographs with the assumed intervals (in this case, every 6 s) during the tests. The obtained images were processed in GOM Software [83] after each test. In the DIC analyses, the following parameters were used: grid size, 57–83 pixels; and grid overlapping, 11 pixels. As a result, the distributions of displacements and strains were obtained on the investigated surfaces.

## 4. Stress Concentration Factor

The cutouts of various shapes, such as circular, rectangular, triangular, etc., often occur in the structural elements [16,17,18,19,20,21,22,23,24,25,26,27,28,29,30,32]. They may be used to reduce the weight of the object or to provide access to a certain part of the device or structure during its operation. The occurrence of such cutouts results in localized high-stress concentration in the surroundings of the hole [33,34,35,67,76]. The highest stresses generally occur at the edge of the hole. Such effect may strongly decrease the strength of the structure, mainly in the regime of the fatigue loadings. The effect of stress concentrations is described by the stress concentration factor (SCF) *K_t_* and calculated as the ratio of the maximal stress at the hole vicinity σ_notch,MAX_ to the nominal stress σ_notch,NOM_, which is calculated with the well-known formulae as follow:

(1) $$K_{t}=\frac{\sigma_{\mathrm{notch},\mathrm{MAX}}}{\sigma_{\mathrm{notch},\mathrm{NOM}}}$$

For all considered samples, the value of the stress concentration factor was estimated using finite element method and Equation (1). In certain cases (a circular hole), detailed theoretical solutions are available [17,19,70,76] and to a large extent agree with FEM results. Unfortunately, in most commonly appearing notches such as openings, only the analytical solutions are given for elements with infinite width (i.e., rectangular [17,18,68] and triangular [71]). It was observed that the stress concentration factors for a plate with finite width estimated using FEM calculations are significantly lower than SCFs for infinite plates. The discrepancy between the theoretically calculated SCFs and their numerical estimations become larger and larger with the decrease of the specimen width (while keeping the constant size of the hole). The obtained differences between such two SCFs approximations reach in some cases even 80% (see Sample 5).

The values of SCFs can be identified for many practical applications with the use of the analytical formulas or with the use of graphical charts given in engineering handbooks [19,76]. Such solutions are generally based on the theories developed by Kirsch [33], Kolosov [34], Mushkelisvilli [69], Lekhnitskiy [68], Neuber [35], and Savin [17]. In the case of a thin panel with a circular hole at the center subjected to the in-plane tension, the SCF can be calculated with the use of the formula proposed by Howland [70]:

(2) $$K_{t}=2.000+0.284\left(1-\frac{d}{W}\right)-0.600{\left(1-\frac{d}{W}\right)}^{2}+1.320{\left(1-\frac{d}{W}\right)}^{3}$$

where *d* is a diameter of the circular hole and *W* is a panel width. For infinity panel, *K_t_* is equal 3. Good agreement of the above solution was confirmed by many analytical and experimental studies [19]. There are also available solutions for SCFs for more complicated cases of elements with circular and elliptical holes, such as eccentrically located in plate circular holes, circumferentially reinforced circular holes, or various elements subjected to bending, bi-axial tension, etc., [19,76].

**Figure 5:**
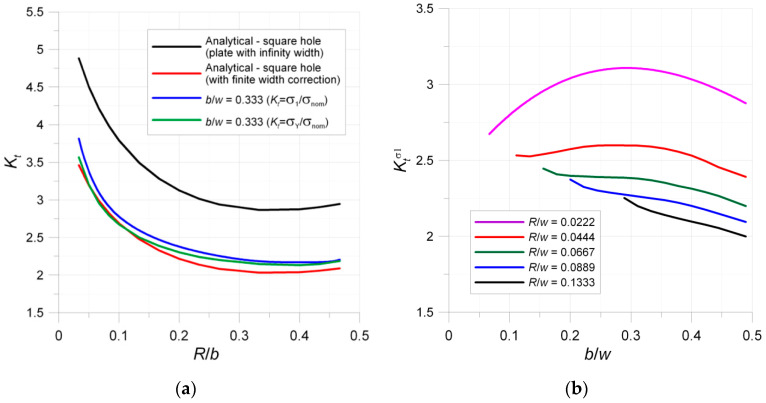
Analytically and numerically (FEM—ANSYS) calculated SCF for panels with square holes (*a* = *b*) with rounded corners subjected to in-plane tension: (**a**) influence of fillet radius—ratio *R*/*b* for constant *b*/*w*; and (**b**) influence of hole size (ratio *b*/*w*) and radius of rounded corner (ratio *R*/*w*) on stress concentration factor.

The remaining two investigated examples with rectangular (square) and triangular holes with rounded corners are more complicated in the analysis. In the case of a rectangular hole with rounded corners subjected to in-plane tension, there are available mathematical or approximate experimental results for infinite panels [14,17,18,19,20,68,72,73]. In the presented study, the following analytical formulae were used for determination of SCF for infinity panel subjected to uniaxial tension:

(3) $$K_{t}^{INF}=C_{1}+C_{2}\left(\frac{b}{a}\right)+C_{3}{\left(\frac{b}{a}\right)}^{2}+C_{4}{\left(\frac{b}{a}\right)}^{3}\;C_{1}=14.815-22.308\sqrt{R/b}+16.298\left(R/b\right),\;C_{2}=-11.201-13.789\sqrt{R/b}+19.200\left(R/b\right),\;C_{3}=0.2020+54.620\sqrt{R/b}+54.748\left(R/b\right),\;C_{4}=3.232-32.530\sqrt{R/b}+30.964\left(R/b\right),\;\mathrm{valid}\;\mathrm{for}\;0.05\leq R/b\leq 0.5\;\mathrm{and}\;0.2\leq b/a\leq 1.0$$

where *R* is a radius of the filleted corner, *b* is the size of a hole measured in the direction parallel to tension, and *a* is a width of the hole in the direction perpendicular to tension. However, the application of the above solution for elements with finite width leads to the high overestimation of SCF. This can be observed in the diagram in Figure 5a with FEM and analytical solution (Equation (3)) for panels with square holes with rounded corners subjected to in-plane tension.

The FEM calculations of SCFs were performed by the authors in ANSYS 19R3 with the use of fine mesh and plane finite elements PLANE182 and linear elastic material behavior. Two different SCF curves were determined with the use of numerically calculated: (1) principal stress σ_1_; and (2) vertical stress σ_Y_ (Y is the direction of tensile load). Both dependencies are presented because the direction of the principal stress σ_1_ is rotated with respect to the tension load when moving along the hole edge. It was observed that, for both rectangular and triangular filleted holes, the maximum of the stress does not exactly appear in the point where the arc of the fillet starts but is slightly moved along the arc. These observations confirmed previous investigations [25,29,30,31]. The results of the numerical investigations mean that the solution for infinite panel has limited application in typical engineering purposes.

The option in the case of finite panels is the use of the FEM solutions [21,22,23] or the finite-width correction factor [19]. The value of the finite-width correction factor is given in Table 4 and the SCF can be calculated as:

(4) $$K_{t}=K_{t}^{\mathrm{INF}}\cdot C_{f}\left(1-\frac{b}{W}\right)$$

where $K_{t}^{\mathrm{INF}}$ is given in Equation (3).

**Figure 6:**
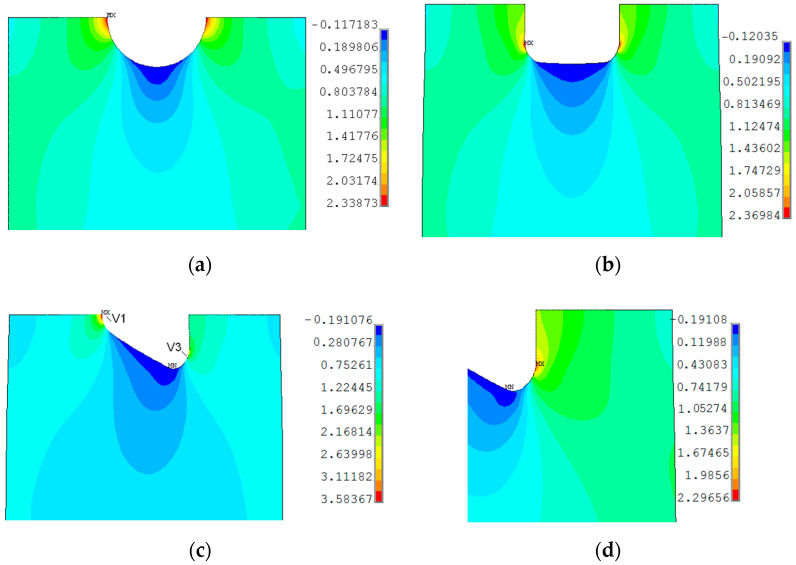
Stress concentration factor for specimens made of S235JR with: (**a**) circular hole; (**b**) square hole, fillet radius R5; (**c**) triangular hole, Corner V1; and (**d**) triangular hole, Corner V3.

**Figure 7:**
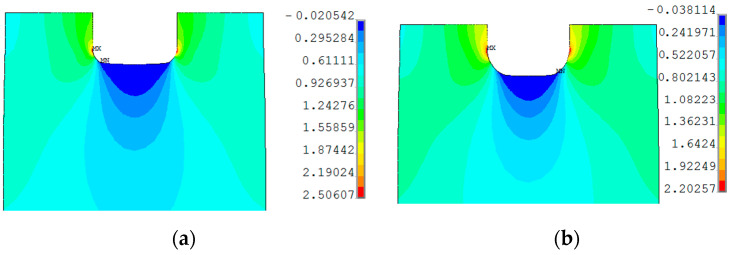
Stress concentration factor for specimens with square holes and rounded corners: (**a**) fillet radius R2; and (**b**) fillet radius R4.

The application of the finite-width correction factor improves the accuracy of the analytical model (Equation (3)); however, the value of the SCF is usually underestimated in relation to the FEM solution. For small ratios *R*/*b* and *a*/*W*, relative error was smaller than −10%. For a larger ratio, mainly when *a*/*w* ≈ 0.5, the error may achieve even −20%. A more complicated situation appears in the case of example with a triangular hole with rounded corners (Figure 1b and Figure 3c). There are only limited analytical solutions for infinite panels [17,19,68,71] with a differently oriented triangular hole. In such a situation, the best solution is the calculation of SCF with the use of the FEM. The values of the SCF were calculated with the use of FEM for all tested samples (Figure 1 and Figure 2 and Table 3). The calculations were made for loads at which nominal stress in the weakened cross-section was equal to 1 MPa. In such a situation, the presented maps (Figure 6 and Figure 7) can be regarded as the contour maps of the SCF or distribution of the vertical σ_Y_ stress after multiplication by the nominal stress. In presented examples in Figure 6 and Figure 7, the SCF was calculated with the use of the vertical σ_Y_ stress. It should be noted that, in the case of the rectangular and triangular (Corner V3; see Figure 6c,d) holes, the major principal stress σ_1_ is rotated with respect to the vertical σ_Y_ stress. This leads to the different values of SCF calculated with the use of σ_Y_ and σ_1_ stresses. Due to the symmetry conditions, the results are presented for half or quarter-part of the investigated specimens. The results of theoretical and numerical calculations of the values of the stress concentration factors for the investigated specimens as well as for infinity plates are summarized in Table 5.

## 5. Experimental Results

The influence of the ratios *b*/*w* and *R*/*w* on the SCFs is given in Figure 5b. In the investigated examples, the value of the corner radius has a stronger influence on the SCF than the hole dimensions. Generally, higher *R*/*w* and *b*/*w* ratios lead to the smallest SCF. Increase of the *R*/*w* ratio results in decreasing in the SCF value. For ratios *R*/*w* higher than 0.0667, the increase of *b*/*w* has a beneficial influence on the SCF value. However, for small *R*/*w* ratios (0.0444 and lower), the trend of the SCF curve is changing. There can be found a suboptimal *b*/*w* ratio for which the SCF achieves its highest value (i.e., *R*/*w* = 0.0222 and *b*/*w* = 0.3). Moreover, in contrast to the other cases, for *R*/*w* = 0.0222, the optimal SCF was achieved for the smallest possible ratio *b*/*w*.

The stress concentration factor *K_t_*, used for determination of the peak of the stress at the notch, fulfilled its function effectively for static loads. Unfortunately, *K_t_* usually gives overestimated results, such as the number of cycles to damage, for fatigue problems [84,85,86]. Following that observation and on the basis of a vast number of experimental tests, another factor was introduced. This is the fatigue notch factor *K_f_*, which is defined as the ratio between un-notched fatigue limit to the notch fatigue limit in the analyzed sample with a notch. *K_f_* is usually lower than *K_t_*, and the difference increases with the decrease of the characteristic size of the considered notch (also the depth or the radius of the notch, etc.). Such discrepancy is explained by the plastic behavior of the material in the notch or by the stress field theory [87,88,89]. It was proved, in a series of tests, that not the peak of the stress in the notch but the average stress in small damage zone around the notch determines the fatigue strength. Another explanation concerns the material with elastic–plastic mechanical properties. If in the sample, certain zones of plastification exist than the peak stress at the notch is reduced in comparison to that one predicted with *K_t_*. Next, the difference between *K_t_* and *K_f_* values exist due to the chemical composition and heat treatment applied for the steel used for the element. In the case of mild steel, the discrepancy is bigger than in the case of high strength and alloy steels. For the same geometry, notch type, and applied load and boundary condition, the zone of damage is lower in case of high strength steel application and it increases the average stress, which lowers fatigue endurance.

Based on empirical tests simple relation between *K_t_* and *K_f_* was proposed [90]:

(5) $$K_{f}=1+q\left(K_{t}-1\right)$$

Such a formula is commonly used in engineering calculations. Here, *q* is known as the notch sensitivity factor and its value varies from 0.0 to 1.0. In the case of fully notch sensitive materials, *q* is equal to 1.0, while, for the notch insensitive materials, it drops down to 0.0. For a particular estimation of *q* values, several proposals have been introduced [19,35,74]. These are empirical in nature and generally depend on the shape of the structural element, the notch type, its radius, the grain size of the material used, and type of loading. At the same time, another concept was established, which is given in [91]. It takes into account the relative stress gradient in the notch area and adopts certain material constant dependent on the yield value limit. Both formulations, for *q* evaluation, are given in many handbooks of fatigue design and are still in use in practical use [92].

### 5.1. S235JR + N Structural Steel

#### 5.1.1. Tensile Tests of Un-Notched Samples

**Figure 8:**
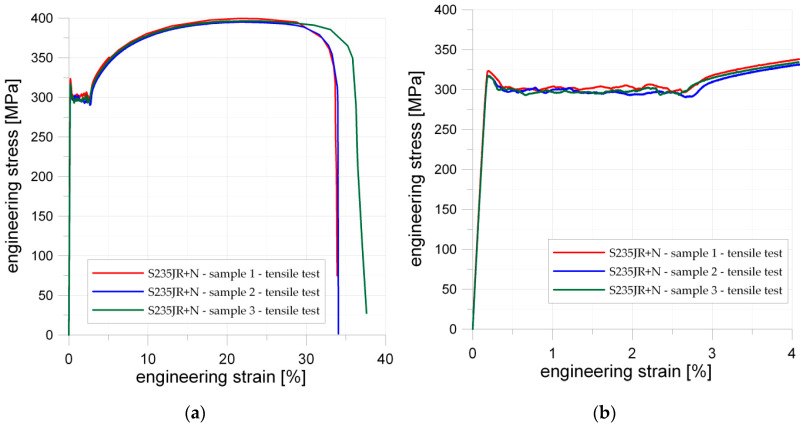
Tensile behavior of S235JR + N: (**a**) engineering σ–ε curves for tested specimens; and (**b**) engineering σ–ε curves for tested specimens-magnified initial part of the curve with elastic and plastic behavior of material.

The mechanical properties of S235JR + N, as well as the tensile σ–ε curves, were determined with the use of the three samples shown in Figure 1a. The geometry of samples was in accordance with Standards [82]. The engineering stress–strain σ–ε curves obtained from the experimental tests are presented in Figure 8. As can be seen, the good repeatability of the results was observed during the three tests. The mechanical properties of S235JR steel after hot-rolling are as follows: upper yield strength *Y_eH_* = 380 MPa and tensile strength *R_m_* = 472 MPa. The determined for normalized S235JR + N steel upper yield strength *Y_eH_* varied within the range 317–323 MPa while the lower yield limit *Y_eL_* varied from 294 to 299 MPa (Figure 8b). The maximal registered in test engineering stress reached almost 400 MPa. Moreover, S235JR + N exhibits considerable yield plateau at the value of about 300 MPa and relatively high strain hardening (see Figure 8). The obtained stress–strain curves are in good agreement with typical material behavior for such materials [93]. These results (Figure 8) were used to set up the geometry of the wide specimens with notches and for modeling material data applied in finite element analysis.

#### 5.1.2. General Information about Experimental Study

**Figure 9:**
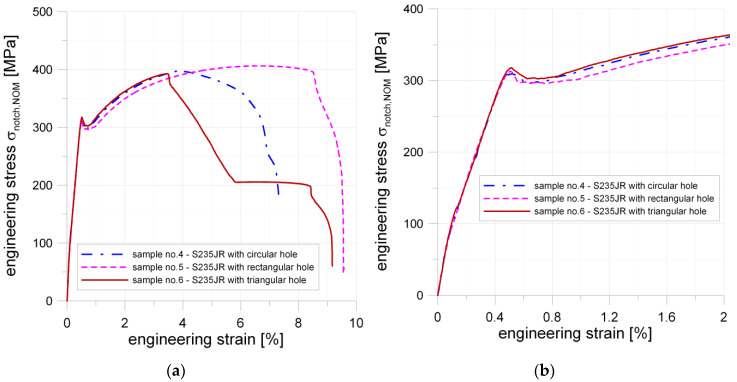
Tensile behavior of S235JR + N: (**a**) engineering σ–ε curves for tested specimens; and (**b**) engineering σ–ε curves for tested specimens–magnified initial part of the curve with elastic and plastic behavior of material.

The influence of the notch geometry on the behavior of structural elements made from S235JR + N steel was studied for three different notches—circular, square, and triangular (Samples 2–4; more details in Table 3). The tension curves obtained for the investigated samples with notches are presented in Figure 9. The results of the experimental tests are given in the form of the engineering σ–ε relationship (see Figure 9a,b). The strong influence of the notch presence and its shape was observed during the performed tests.

To understand the phenomena occurring in the investigated specimens with circular, square, and triangular notches subjected to tension, the following stresses have been considered:

(1) Nominal stress in non-weakened cross-section—$\sigma_{\mathrm{NOM}}=F/wt$;
(2) Nominal stress in cross-section weakened by a notch—$\sigma_{\mathrm{notch},\mathrm{NOM}}=F/\left(w-l\right)t$; and
(3) The estimated maximal stress in cross-section weakened by a notch—$\sigma_{\mathrm{notch},\mathrm{MAX}}=\sigma_{\mathrm{notch},\mathrm{NOM}}\cdot K_{t}$.

**Figure 10:**
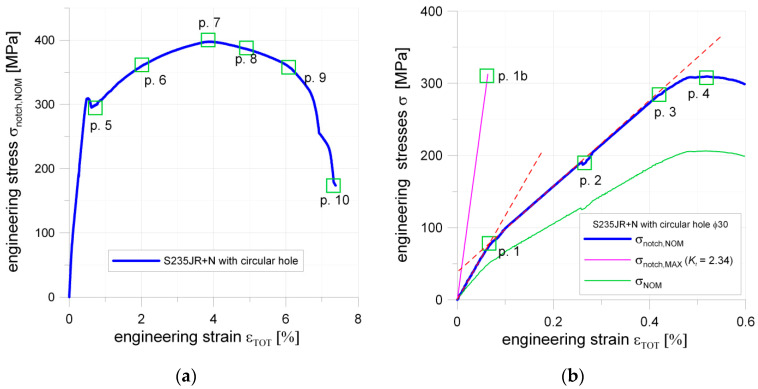
Tensile behavior of S235JR + N: (**a**) engineering σ–ε curve for plate with circular hole; and (**b**) magnified initial part of the curve.

The estimated maximal stress in a weakened by a notch cross-section was calculated by multiplying nominal stress in cross-section weakened by a notch by a stress concentration factor *K_t_*. The values of *K_t_* are given in Table 5. The presented diagrams are plotted as functions of stress related to the cross-section weakened by a notch—σ_notch,NOM_ = f(ε_TOT_). In the first part of the σ–ε curves, for the nominal stress σ_notch,NOM_ in the range 0–300 MPa, the particular curves can be interpolated by two linear functions. This effect is caused by the appearance of the first plastic strains around the notches. In the σ–ε curve, certain characteristic points are spotted. For example, the points in which plastic strains occur (marked as Point 1; see Figure 10b) were defined by the intersection of interpolating lines.

#### 5.1.3. Plate with Circular Hole

**Figure 11:**
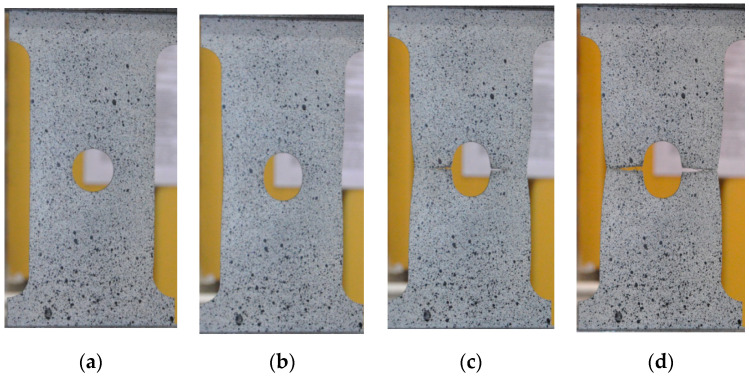
Tensile behavior of plate with circular hole made of S235JR + N: (**a**) Point 4; (**b**) Point 7; (**c**) Point 9; and (**d**) Point 10. The points are given in Figure 10.

In the case of the sample with the circular notch, the estimated maximal elastic stress in a notch was about σ_notch,MAX_ = 305 MPa (Figure 10b; Point 1b). It is in good agreement with material behavior given in Figure 8 (the determined minimum yield limit from the tensile test was equal to *Y_eL_* = 294 MPa). In the case of the plate with the circular notch, a slight decrease of strength was observed for the nominal stress σ_notch,NOM_ = 188 MPa; Point 2). The σ = f(*ε*_TOT_) relationship was no longer linear when σ_notch,NOM_ exceeded 284 MPa (Point 3 in Figure 10b). Full plastification in the weakened cross-section occurred for σ_notch,NOM_ = 309 MPa (Point 4 in Figure 10b and Figure 11a). This effect was also confirmed by the DIC analyses (Section 6). Continuing the tension of the sample the point of the maximal tension is reached. This is Point 7 (Figure 10a), and, up to this point, no traces of cracks were observed. However, the narrowing of the investigated sample was already visible (Figure 11b). After crossing Point 8, the crack initiation along both sides of the opening started. At Point 9, the length of cracks reached approximately 50% of the width of the weakened cross-section (Figure 11c) and the chart line breakdown was observed on the tension curve (Figure 10a). After that, Point 10 was reached when the total fracture of the sample took place (Figure 11d).

#### 5.1.4. Plate with Square Hole

**Figure 12:**
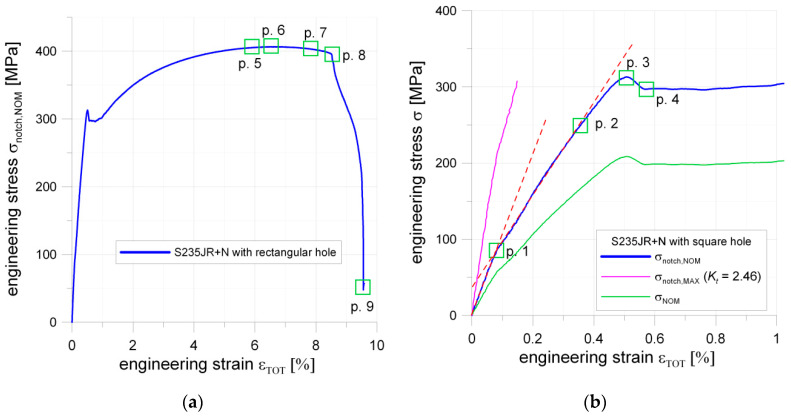
Tensile behavior of S235JR + N: (**a**) engineering σ–ε curve for plate with square hole with corner fillet radius; and (**b**) magnified initial part of the curve.

In the case of the plate with the square hole with fillet corners, the first bend of the curve is observed at Point 1 (Figure 12b; Point 1). This can be attributed to the appearance of the first plastic deformations observed in the notch area. The calculated with the use of *K_t_* and evaluated by FEM the maximal elastic stress in the notch was about σ_notch,MAX_ = 219 MPa. This value was significantly lower than the yield limit of the material obtained in a standard tension test. The cause of this phenomenon was revealed during the DIC analyses, in which it was observed that slight non-symmetrical tension occurred during the tests (the probable cause was the unexpected looseness of the screw joints fastening the tested plate in grips). In the investigated sample, the theoretical value of the maximum stress was closer to the observed one (plastification for 290 MPa). The hole manufacturing technology—water jet cutting—can be responsible for that, namely, a non-smooth transition between the straight part, and filleted corner was observed, which was the source of the future crack. At Point 2, the curve became no longer linear; this was for σ_notch,NOM_ = 248 MPa.

**Figure 13:**
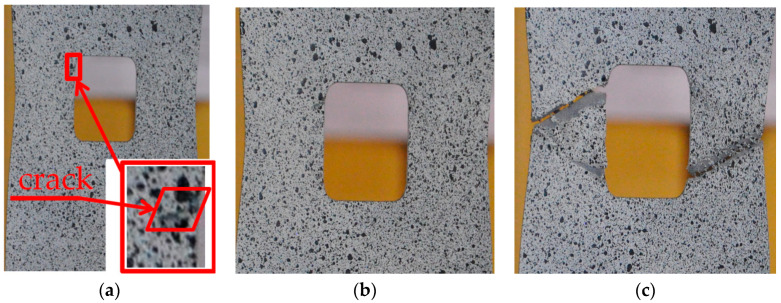
Tensile behavior of plate with square hole made of S235JR + N: (**a**) Point 5 with magnified zone with crack; (**b**) Point 6; and (**c**) failure. The points are given in Figure 12.

This phenomenon happened earlier than in the case of the circular hole (the same net cross-section, loading condition, and sample thickness). The probable source of this was the presence of two severe notches on each side of the hole. At Point 3 (Figure 12b), the local maximum appears, which was connected with full plastification of the weakened cross-section (σ_notch,NOM_ = 313 MPa), and after that characteristic slight drop in stress value (like during the tension of standard non-weakened sample) appeared. During the further part of the test, the plastic flow took place along with the material hardening. In this way, Point 5 was reached, where the first crack on the edge appeared (Figure 12a and Figure 13a). Shortly after, the load reached its maximum (Point 6; Figure 12a and Figure 13b). Further increase of the displacements caused that at Point 7 two cracks were present and their length raised to 50% of the width of the weakened cross-section, shortly after that at Point 8 one side of the sample was completely fractured and disastrous development of the crack length was observed on the opposite side of the square hole (Figure 13c).

#### 5.1.5. Plate with Triangular Hole

**Figure 14:**
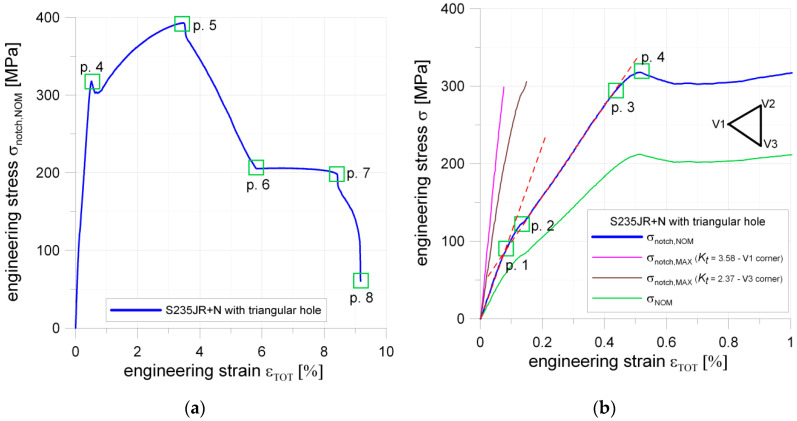
Tensile behavior of S235JR + N: (**a**) engineering σ–ε curve for plate with triangular with corner fillet radius; and (**b**) magnified initial part of the curve.

**Figure 15:**
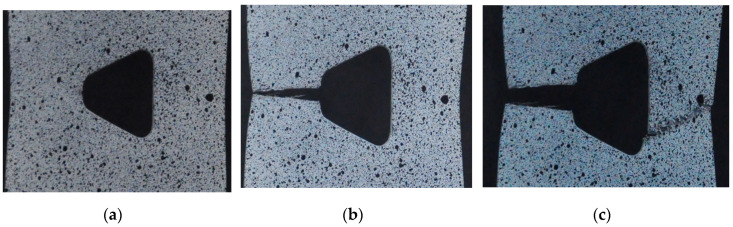
Tensile behavior of plate with triangular hole made of S235JR + N: (**a**) Point 5; (**b**) Point 6; and (**c**) failure. The points are given in Figure 14.

The sample with the triangular notch (illustrated in Figure 14) exhibits different behavior when compared with samples with circular and square notches. The first bend of the plot is observed at Point 1 (Figure 14b), which corresponds to the plastification observed in vertex V1. For this point, the estimated with the use of the numerically calculated *K_t_* maximal vertical stress in notch reached σ_notch,MAX_ = 293 MPa. The further increase of the external load caused the plastification in vertex V2 and V3. The estimated vertical stress values for these notches were equal to σ_notch,MAX_ = 290 MPa. At Point 3, the plot became no longer linear (approximate value of nominal stress in weakened cross-section σ_notch,NOM_ = 295 MPa). Further behavior of the sample is determined by the combination of elastic and plastic behavior. The full plastification of the sample is reached at Point 4, for which the nominal stress value was equal to σ_notch,NOM_ = 318 MPa (Figure 14). The first observed crack was spotted on the side with Vertex 1, for which the total strain reached the value of about 2.7% (it corresponded to σ_notch,NOM_ = 380 MPa approximately). The maximum load resulted in maximum stress equal to σ_notch,NOM_ = 393 MPa, which was reached at Point 5 (Figure 14a and Figure 15a). After that, the initiation of the crack was observed, and its rapid development took place. Finally, the partial damage of the sample appeared (Point 6; Figure 15b), namely on the side of the Vertex 1. At Point 7, a crack was formed in the vicinity of Vertex 3. The appearance of this crack rapidly resulted in total damage of the investigated sample (Figure 15c).

### 5.2. S355MC Microalloyed Fine-Grain Structural Steel

**Figure 16:**
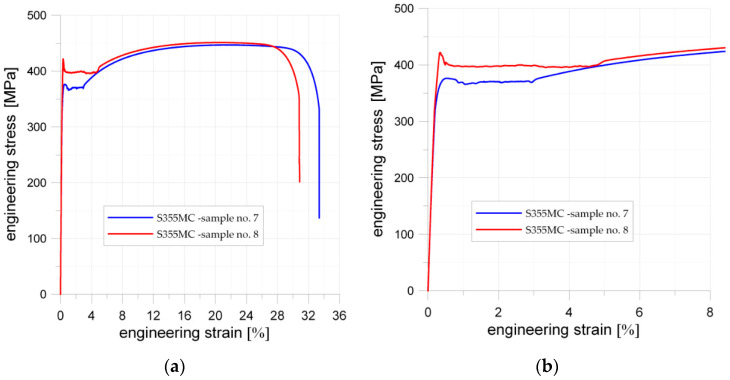
Tensile behavior of S355MC: (**a**) engineering σ–ε curves for tested specimens; and (**b**) engineering stress–strain curves for tested specimens—enlargement of the curve with elastic and plastic behavior of material.

Similar, typical static tensile tests were performed for two specimens with dimensions given in Figure 2a. Here, the tested material was the microalloyed fine-grain structural steel S355MC. The sheet of metal was manufactured by thermo-mechanical rolling. To determine the anisotropy of the material, the samples were cut from the sheet in perpendicular (Sample 7) and parallel (Sample 8) directions to the rolling direction. The yield limit and tensile strength were higher in both cases than the minimum values given in the standards (see Table 2). However, the manufacturing process substantially influences the mechanical properties of the material. The sample cut in the parallel direction to the rolling direction (Sample 8 in Figure 16) showed higher mechanical properties than sample cut in the perpendicular direction to the rolling (Sample 7), namely higher upper yield strength (Sample 7, *Y_eH_* = 376 MPa; Sample 8, *Y_eH_* = 422 MPa), a larger difference between higher and lower yield strength, and larger percentage yield point extension, whereas the tensile strength for both samples was almost the same (Sample 7, *Y_eH_* = 446 MPa; Sample 8, *Y_eH_* = 451 MPa).

**Figure 17:**
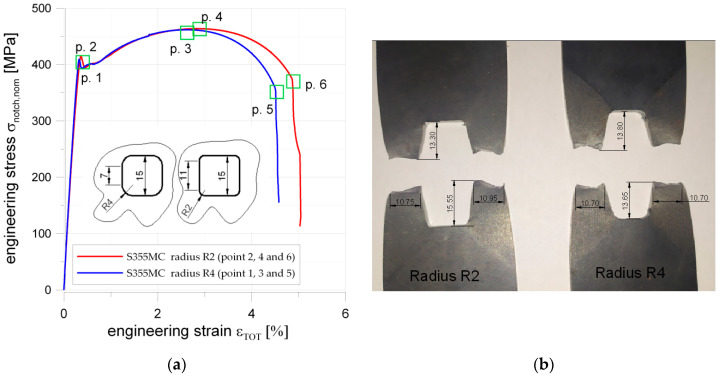
S355MC with rectangular hole: (**a**) engineering stress–strain curves for tested specimens and details of hole; and (**b**) failed samples made of S355MC with square hole with fillet radius R2 and R4.

The influence of the stress concentrators was tested with the use of two samples with a square hole with filleted corners each. The dimensions of the square hole were the same in both specimens 15 mm × 15 mm. The different fillet radii were applied: 2 or 4 mm (see Figure 2b). The results of static tensile tests are given in Figure 17a. The sample with smaller edge fillet radius (R2) have slightly higher upper yield limit (Points 1 (σ_notch,NOM_ = 409 MPa) and 2 (σ_notch,NOM_ = 415 MPa); more details in Figure 17a) and tensile strength (Points 3 (σ_notch,NOM_ = 462 MPa) and 4 (σ_notch,NOM_ = 464 MPa); Figure 17a). Interesting in this case was also that the increase of the corner radius (from R2 to R4) of the square hole resulted in smaller total strains for the yield limit and tensile strength. Both samples were damaged in the mid-side cross-section of the weakened by hole part. The sample with a larger radius (R4) was destroyed for essentially smaller elongation (see Points 5 (R4) and 6 (R2)). This means that, under the static tensile load, increase of the fillet radius may substantially deteriorate sample strength. However, the FE analyses demonstrate that an increase of the radius decrease SCF (see Figure 7 and Table 3) and provides higher strength. This effect under static tensile load can be explained by behavior of the weakened cross-sections and low sensitivity of the S355MC steel for notch presence. The difference in geometries of the samples is that in the case of the smaller fillet radius (R2) the length of the straight part of the hole is 11 mm, while, in the case of the fillet radius equal to 4 mm, it goes down to 7 mm (see Figure 17a). The longer side length allows for the development of higher elongation of the weakened part of the sample under elastic-plastic loading conditions. This is confirmed in Figure 17b: the vertical distance between the opposite sides is bigger in case of a smaller fillet radius. Low sensitivity of the S355MC steel for notch presence is also visible due to the only slight differences in the σ–ε shapes for different *K_t_* (2.25 for R4 and 2.59 for R2); no clear bends of σ–ε curves in the range below the plasticity in the whole cross-section is observed. Moreover, the values of σ_notch,nom_ for yield limit and tensile strength are consistent with the limits obtained for samples without notches (see Figure 16), while the maximal stresses in notches in Points 1 and 2 may achieve σ_notch,MAX_ = 920 MPa for *K_t_* = 2.25 and σ_notch,MAX_ = 1075 MPa for *K_t_* = 2.59, respectively. It means that the presence of notches does not reduce the strength of the sample under static loading, as was visible for S235JR + N steel. It should be also noted that the S355MC steel did not show sensitivity to laser cutting. It can be attributed to low carbon content (0.07%) and then no elements increasing hardenability are present in the microstructure and there is no risk of tough and brittle carbides formation.

These examples demonstrate that commonly used assumptions—i.e., larger radius leads to larger strength—may not always be right and are influenced by the notch sensitivity. To confirm this phenomenon, further studies will be performed.

### 5.3. 41Cr4 (40H) Alloy Structural Steel (Toughening Steel)

**Figure 18:**
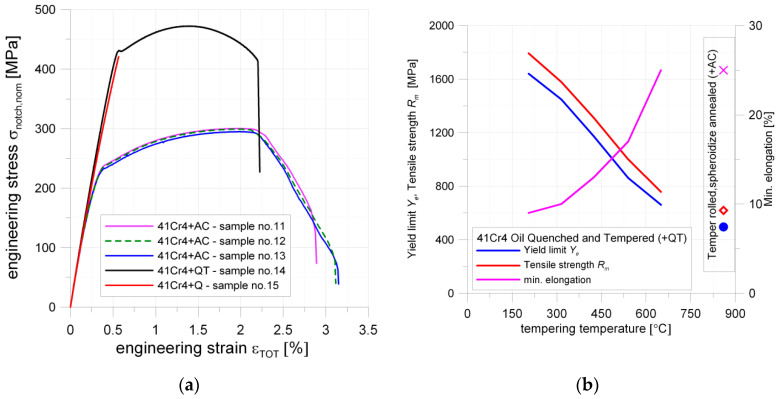
Steel 41Cr4: (**a**) Stress–strain curves for specimens made of 41Cr4 with square hole with fillet radius R2 (AC, spheroidized annealed; QT, quenched and tempered; Q, only hardened); and (**b**) minimal mechanical properties of 41Cr4 steel after heat-treatment.

**Figure 19:**
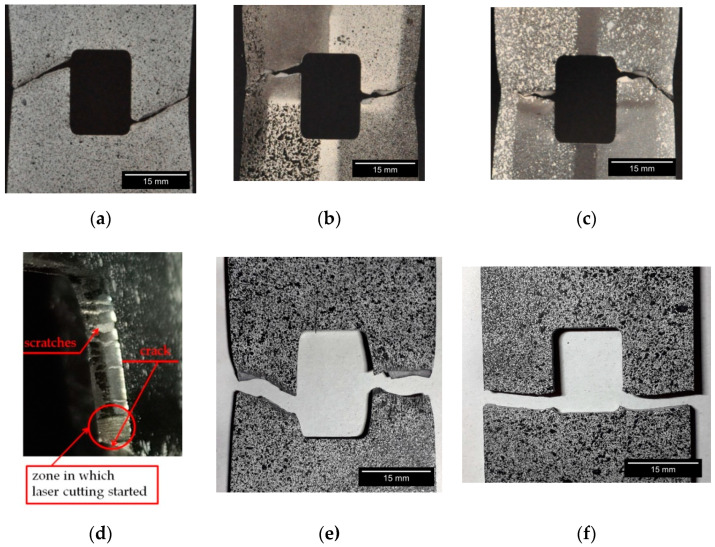
Failed samples made of 41Cr4: (**a**) Sample 11, 41Cr4 + AC, 23 HRC; (**b**) Sample 12, 41Cr4 + AC, 23 HRC; (**c**) Sample 13, 41Cr4 + AC, 23 HRC; (**d**) view on lateral side of the hole of Sample 12 with visible scratches, start point of laser cutting and crack; (**e**) 41Cr4 + QT, 35 HRC; and (**f**) 41Cr4 + Q, 54 HRC.

The static tensile tests for 41Cr4 steel were made for samples with a square hole 15 × 15 mm with a fillet radius of 2 mm. The geometries (Figure 2c), loading conditions, SCF for Samples 11–15 were the same. The experimental tests were conducted for materials subjected to the different heat treatments and these appeared to be of major influence on the test results. Samples 11–13 were temper rolled and spheroidized annealed (+AC), Sample 14 was quenched and tempered (+QT) and Sample 15 was only quenched (+Q). All samples made of 41Cr4 revealed smaller ultimate strain (2.2–3.2%) than the sample made of S355MC (5%). Good repeatability of the stress–strain curve was obtained for the spheroidized annealed specimens 41Cr4 + AC (Figure 18a). The influence of heat treatment for 41Cr4 is presented in Figure 18b. It was obvious that, in the first phase of tests, the samples made of the same material and subjected to the different heat treatments exhibit the same slope of the stress–strain curve. The same behavior was also obtained for samples containing stress concentrators, as given in Figure 18a. The failure forms of the notched samples made of 41Cr4 are given in Figure 19.

Notched samples made of 41Cr4 + AC revealed significantly smaller yield limit (reduced about 50%) and ultimate tensile strength than notched samples made of 41Cr4 + QT and 41Cr4 + Q, but a much larger ultimate strain was observed in comparison to hardened or quenched and tempered 41Cr4 samples. However, it should be noted that, despite the smaller strength of spheroidized annealed samples (41Cr4 + AC), the tensile work until failure is significantly higher for normalized samples than for the quenched one (41Cr4 + Q). The cracks in samples made of 41Cr4 + AC were initiated at two points: the first inclined crack at the end of the fillet radius (Figure 19a) and the second horizontal crack, in two cases, at the middle of the side length of the square (Figure 19b,c). The crack initiation in the first point can be explained by the stress concentrations at the notch. The initiation of the crack at the second point is caused by using laser cutting to make square holes. Such cracks (Figure 19b,c) initiated exactly at the end of the zone in which laser cutting started (Figure 19d). The laser cutting in this case caused local structural changes in the material at the edge of the hole. The thickness of the area subjected to local structural changes known as heat affection zone (HAZ) is usually smaller than 0.3 mm [94,95]). Thus, the usage of the laser cutting resulted in the increase of the hardness in the HAZ (high carbon (0.39%) and chromium (1.04%) content; both elements increase hardenability and Cr additionally increases hardening depth) at the edge of the square hole [95,96,97]. Excluding these small hardened volumes of the materials around the holes, the rest of the volume of the samples (Figure 19a–c) were in the softened state. However, such a hardening around edge significantly increases the notch sensitivity of the material. This effect was confirmed by multiple scratches (Figure 19d) observed on both edges of the hole subjected to tension and significant change of the hole shape from square to rectangular one (there is no visible barrel shape; Figure 19a–c).

The notched sample subjected only to hardening 41Cr4 + Q revealed brittle behavior without visible plastic strains in the σ–ε curve. The cracks initiated in the points with the highest stress concentrations and no plastic deformation were observed around the hole area (Figure 19f).

The notched sample subjected to the typical heat-treatment procedure 41Cr4 + QT (hardening and tempering), in the first part of the test, until the yield limit was reached, revealed almost the same slope of σ–ε curve as 41Cr4 + Q. The yield limit of 41Cr4 + QT was similar to the ultimate stress of 41Cr4 + Q. However, it can be observed in Figure 18b that quenched and tempered 41Cr4 steel has significantly reduced mechanical properties such as *Y_e_* and *R_m_* in comparison with the non-tempered or tempered 41Cr4 steel at low temperatures [98]. It means that the yield limit of un-notched and only quenched material should be at least two times higher than for quenched and tempered one at 600 °C. This similarity of the yield limit of notched sample 41Cr4 + QT with the ultimate strength of notched sample 41Cr4 + Q is caused by extreme sensitivity to the notch presence of only quenched material. Further behavior of notched samples made of 41Cr4 + QT is typical for high-strength steel. The cracks were initiated at the same point as for 41Cr4 + AC samples. However, the second horizontal crack (Figure 19e) was not initiated at the point in which laser cutting was started. In this case, the laser cutting had no visible influence due to the hardened state of the material in the whole volume. Due to the larger hardness of this sample, the vertical elongation of the hole was significantly smaller than in 41Cr4 + AC and the shape of the hole at fracture took the form of a barrel.

## 6. DIC and FEM Analyses

The damage caused by external loading can be detected, localized, and identified by the use of the different non-destructive methods. Different techniques based on acoustic emission, lamb wave propagation, or with the use of scanning electron microscopy, thermography camera, Moire interferometry, etc. are in use [45,46]. These methods and their advantages and disadvantages are described in the Introduction. Considering recent possibilities of the computers and high-speed cameras with high resolution, the DIC system seems to be convenient in the application of damage detection and growth [47,48,49,50,51,52,53,54,55,56,57,58,59,60,61,62,63,64,65,66]. With the use of the professional DIC system, it is possible to achieve good quantitative and qualitative calculation of displacements and strains fields over the investigated surface.

In the investigated case, the DIC analyses were performed with the use of the GOM Software. The photographs were made by Nikon D90 camera with constant time intervals (6 s). The main aims of the DIC analyses were:

(1) Detection and control of plastic strain zones growth;
(2) Determination of the possibilities of DIC method in detection of damage and prediction of failure form; and
(3) Verification of the DIC results by FEM analyses.

It is obvious that the applied equipment (average high-resolution camera) does not give the same possibilities as professional DIC systems (the highest resolution, sensitiveness, accuracy, etc.), but such instrumentation is widely available and accurate enough for the presented study. The input data to the DIC analysis were made by making a series of images of the investigated surface coated by randomly distributed speckle pattern. The first image was assumed as a reference one (unloaded sample) and the others were made in constant time-intervals with the increasing tensile load. After the tests, the images were processed with GOM Software [83], in which every picture made during the tensile tests were compared with the reference one. It allows for the determination of strains over the investigated surface. The vertical major strain fields corresponding to the tensile stress were calculated for particular specimens and presented in Section 6.1 and Section 6.2. The all presented images using the DIC analyses show the samples with the full width or half-width of the samples, respectively.

### 6.1. S235JR + N Structural Steel

#### 6.1.1. Sample with Circular Hole

**Figure 20:**
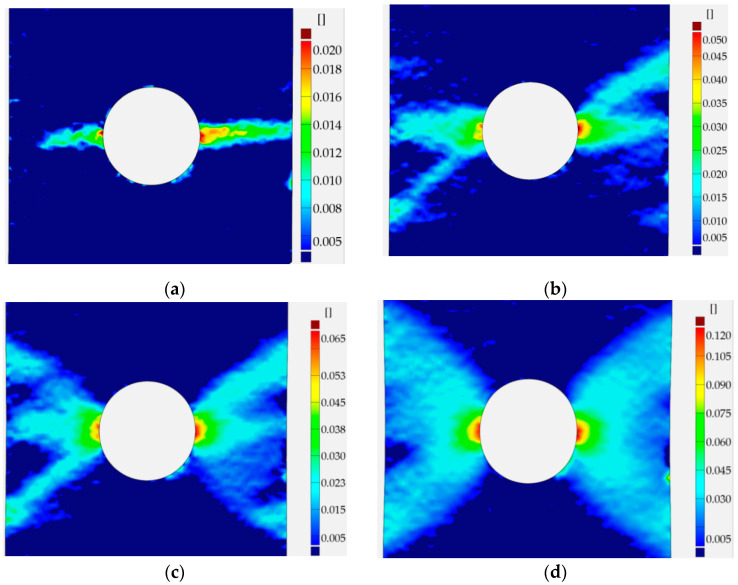

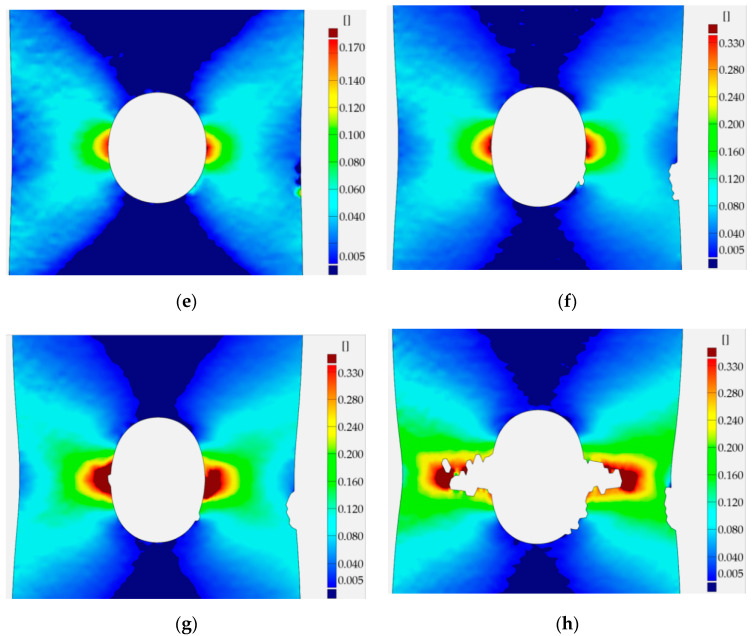
Strains field on investigated surface of plate made of S235JR + N with circular hole calculated with the use of DIC; total vertical displacement u_MTS_ and corresponding vertical ε_TOT_ (in brackets): (**a**) 0.79 (0.44); (**b**) 1.09 (0.61); (**c**) 1.39 (0.74); (**d**) 2,40 (1.34); (**e**) 3.60 (2.00); (**f**) 6.69 (3.72); (**g**) 8.49 (4.72); and (**h**) 10.9 (6.1).

The distributions of the total vertical strain on the surface of the plate with the circular hole for different elongations obtained using the DIC method are presented in Figure 20. The obtained sensitiveness was equal to ε_sensGOM_ = 0.005 for the investigated sample. The first image (Figure 20a), corresponding to Point 3 in Figure 10b, presents the appearance of the first plastic strains, which occur on the opposite sides of the circular hole in the area of the thinnest cross-section of the sample. It is worth noting that the limit value for the elastic strains is ε_elastic_ = 0.0014. The further increase of the external load results in the growth of strains in the area surrounding the hole. The propagation of the plastic deformations to the outer edge of the sample (in the horizontal direction, it is accompanied by the first two inclined, diagonal paths of plastic strains (Figure 20b)). The distribution of strain field presented in Figure 20c corresponds to Point 5 of the σ–ε curve (Figure 10). In this case, the presence of the map of deformations shows the characteristic, symmetric pattern, which is repeated with higher absolute values for higher loads (see Figure 20d–f). After slight strengthening (Figure 20e; Point 6 in Figure 10a), the maximum value of total vertical strain achieves value above 0.330 (Figure 20f, which corresponds to Point 7 in Figure 10a and Figure 11b), which seems to be the starting point for the initiation of cracks. Further development of cracks and plastic zones is presented in Figure 20g (corresponding to Point 8 in Figure 10a; there are cracks on both sides of the circular hole) and in Figure 20h (corresponding to Point 9 in Figure 10a and Figure 11c), in which cracks have the length equal to almost half of the width in the thinnest cross-section. It can be observed that, for all considered values of the applied load, the pictures do not exhibit the full symmetry with respect to the vertical line placed in the middle cross-section. The reason for that is the real material properties in the investigated sample, presence of micro faults, the quality of speckles placed on the surface of the investigated structure, and the accuracy of the applied method overall.

**Figure 21:**
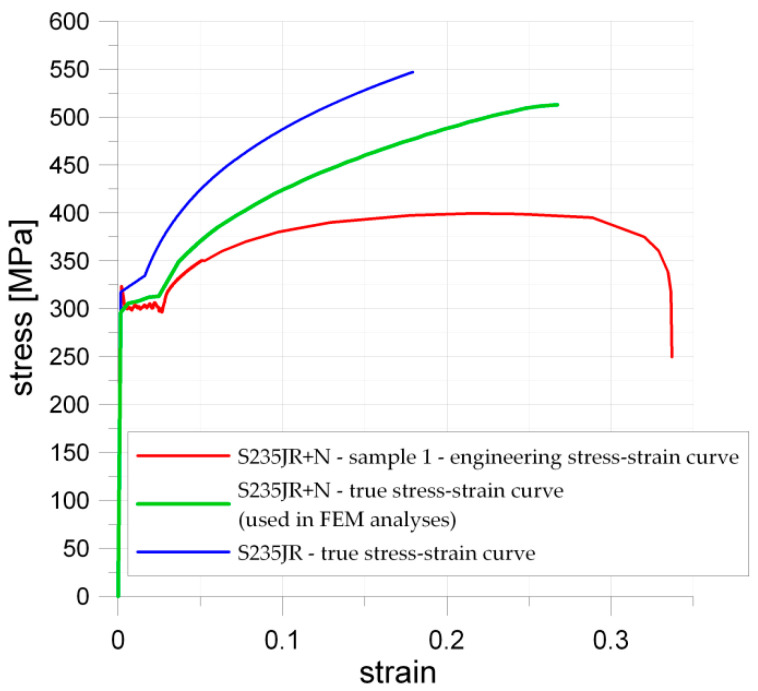
Engineering and true stress–strain curves for S235JR and S235JR + N steels.

In parallel, the respective finite element model was prepared. For that purpose, Ansys software [99] was used. The finite elements for the analysis of plane stress problems were used, and here two choices were possible. The elements PLANE182 with the linear approximation of the displacements within the elements or higher-order elements PLANE183 could be applied. The latter provide a higher level of accuracy of solutions, particularly in case of linear elastic problems. The elements of lower order are usually used in the case of strong non-linearities, such as advanced plastic deformations. Nevertheless, to receive the numerical results of high quality, the relatively dense mesh should be prepared, particularly in the investigated area of stress concentration—the vicinity of the opening. In the performed calculation, only the symmetric half was numerically tested with the symmetry boundary conditions imposed in the vertical line of symmetry. The mesh was generated for the structure symmetric quadrant and after that mirrored for the rest of the analyzed part, which provides the perfect symmetry of the generated mesh. The size of the finite elements was chosen to provide the structural energy error generated by the program below 2% for the elastic analysis, particularly in the vicinity of the opening area. The numerical FEM analyses were performed in the regime of the large plastic deformation. In such a case, the implementation of the true stress–strain material curve into FE analysis is necessary. Such a relationship is based on the instantaneous cross-section shape of the tested sample. The true σ–ε curve for the S235JR steel was determined and proposed by Kossakowski [43,100]. There are also available mathematical formulations for determination true stress and true strains from the engineering σ–ε behavior of material measured during the typical tensile test [14]. However, such relationships are generally valid only up to the point of necking and may result in a quite large error of estimation. The assumed material model was determined based on the measurements of the sample during the tension test and verified by the commonly used engineering—true σ−ε relationships. The comparison of the determined engineering and true σ–ε curves for S235JR + N obtained from the tensile test (i.e., Sample 1) and true curve for S235JR proposed by Kossakowski is given in Figure 21. The σ–ε curve following the results obtained in tension test (see Figure 21) was used in modeling, and the part of the curve corresponding to plastic deformations was modeled using 20 points joined with straight lines, which approximate its real shape (the number of points, which can approximate the stress–strain curve is limited in the program). The options for large deformations and full Newton–Raphson solution procedures [99] were switched on during the analysis. The external load was introduced in the form of the vertical displacements imposed along the bottom edge of the model.

**Figure 22:**
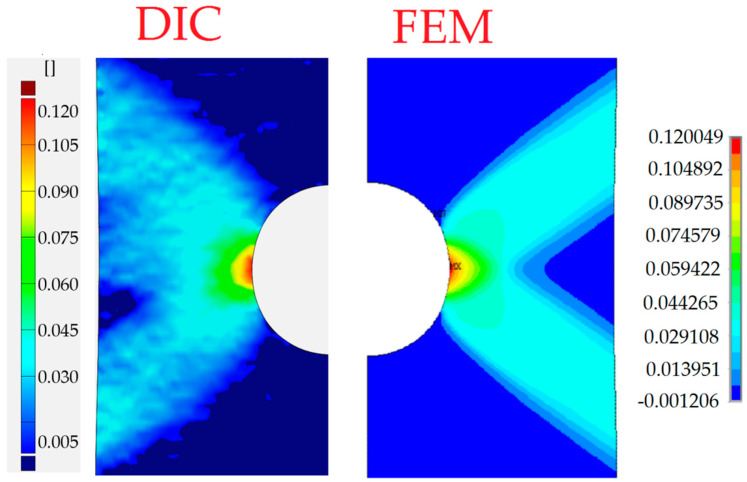
Comparison of total vertical strains distributions.

The distribution of total strains in the vertical direction was chosen for the comparison. In Figure 22, the DIC–GOM results and the Ansys finite modeling (FEM) solution are shown and compared. In this picture, the same levels of the contour lines were set for both types of analysis. As can be seen in Figure 22, the relatively good agreement of the obtained results is observed for the two contour maps.

#### 6.1.2. Sample with Square Hole

**Figure 23:**
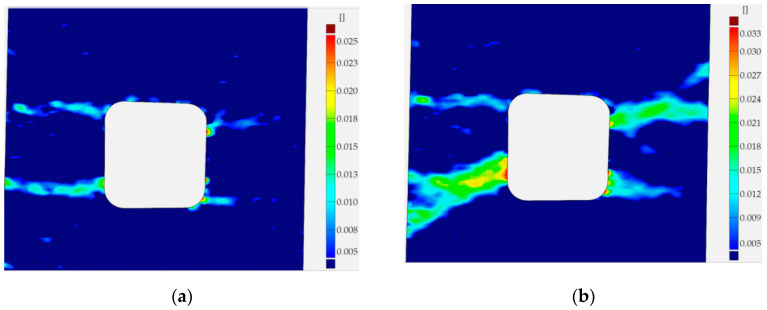

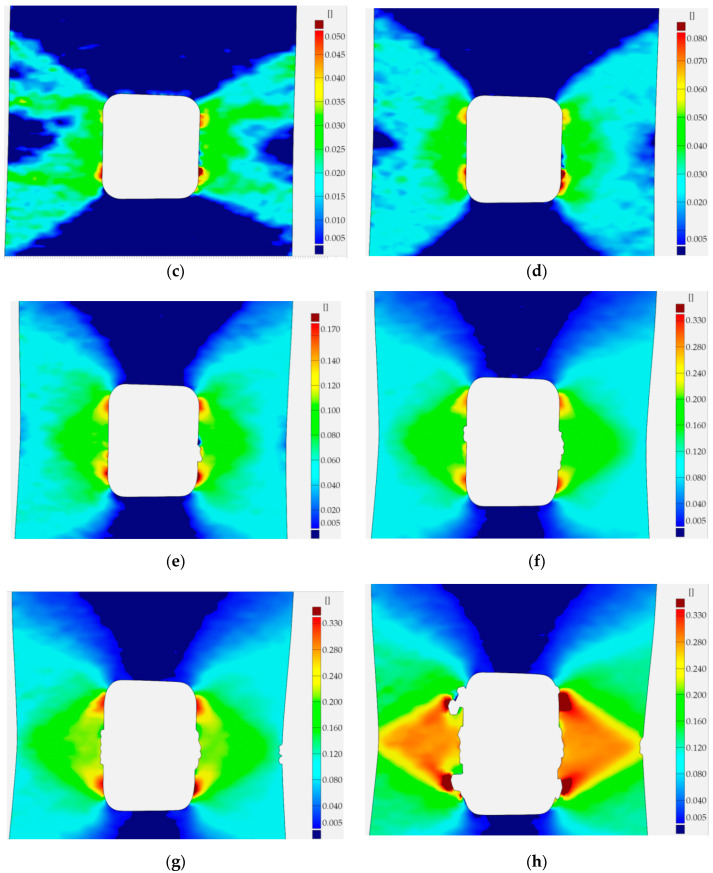
Distribution of the major vertical strains for S235JR + N plate with rectangular hole calculated with the use of DIC; total vertical displacement u_MTS_ and corresponding vertical ε_TOT_ (in brackets): (**a**) 0.74 (0.41); (**b**) 0.93 (0.51); (**c**) 1.57 (0.87) (**d**) 2.42 (1.35); (**e**) 5.59 (3.11); (**f**) 9.59 (5.33); (**g**) 10.26 (5.70); and (**h**) 13.25 (7.37).

A similar study was performed for the structure with the square hole with rounded corners in the center part. Here, the results for the radii 5.0 mm and the distance 30.0 mm between the opposite sides of the square are shown. This means that the thinnest cross-section has the same area as in the previous study. However, the analytical results for the tensed thin plate with such a void result in the higher value of the stress concentration factor than for circular opening. This tendency is kept when going to the finite width of the plate. Figure 23 presents the results of the DIC analysis.

Here, the first plastic strains occur in the surroundings of the fillets in the thinned cross-sections (Figure 23a). Such distribution of strains was achieved for total strain ε_TOT_ = 0.41 (slightly beyond Point 2 in Figure 12b). The further increase of the tensile load leads to an increase of strains and their propagation to the edges of the plate in horizontal and diagonal directions (Figure 23b). This image corresponds to Point 3 of the σ–ε curve (Figure 12b), in which the maximal tension was observed before tensile force drops. Plastification of the weakened transversal cross-section of the plate is also observed in Figure 23b. Further increase of elongation results in the expansion of the plastic zones in a diagonal direction (Figure 23c,d). The high plastic strains are concentrated in the form of zones of triangular shape along hole edges (Figure 23d–f). The maximal strains are observed in the four points around the square edge in points where straight vertical edge turns into the arc (Figure 23e,f). The initiation of the cracks appears when the total vertical strain exceeds ε ≈ 0.33 (Figure 23f). It should be noted that the first cracks on photographs were observed on the image corresponding to Point 5 of the σ–ε curve (Figure 12a, Figure 13a and Figure 23f). Propagation of such cracks leads to the further increase of the plastic strains in diagonal directions (Figure 23g) and this is observed while keeping the same maximum value in the legend of the respective contour maps. The last image (Figure 23h) presents the total vertical strain distribution when cracks achieved 50% of their maximal length (Point 7 in Figure 12a). In this case, DIC analysis detected two cracks on the left-hand side. This was incompatible with the observed failure form given in Figure 13c, in which it can be seen that there was no crack at the bottom corner of the left-hand side of the hole. This can be explained by the thinning of the sample in the surrounding of the notch by about 25% and in the peeling of the varnish layer from the tested surface (see Figure 13c).

**Figure 24:**
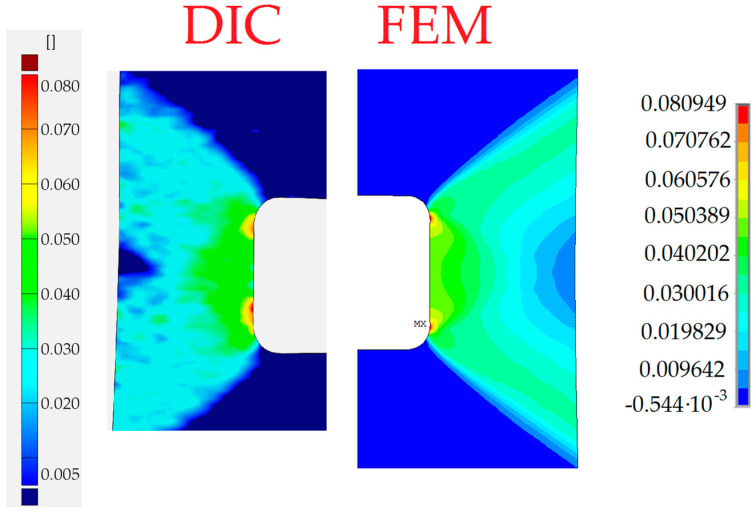
GOM–ANSYS comparison of total vertical strains distributions.

The finite element mesh was prepared in a similar way as for the sample with the circular opening. The criterion for the choice of the element size was the same as in the previous case—the sample with the circular hole. As can be seen in Figure 24, the fairly good agreement of the obtained results is again observed for the two compared contour maps.

#### 6.1.3. Sample with Triangular Hole

The final study for S235JR + N was performed for the plate with the triangular hole in the center part. The opening was cut with rounded corners with radii equal to 5.0 mm. The analyses of the vertical strains distribution for this plate and comparison of them with strains distributions for plates with circular and rectangular hole revealed the following conclusions:

(1) On the V1 corner side: The shape and growth of the plastic zones is similar as for plate with the circular hole.
(2) On the opposite side (Corners V2 and V3): The spread of the plastic zones is qualitatively similar to that one observed in the plate with the square hole.

**Figure 25:**
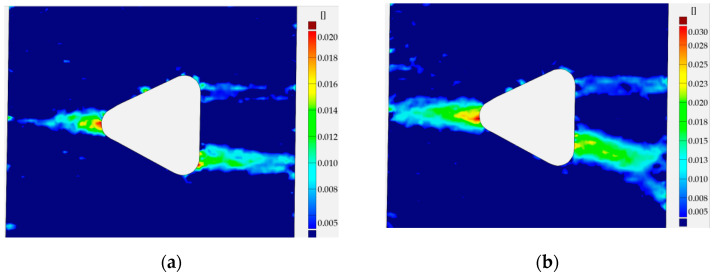

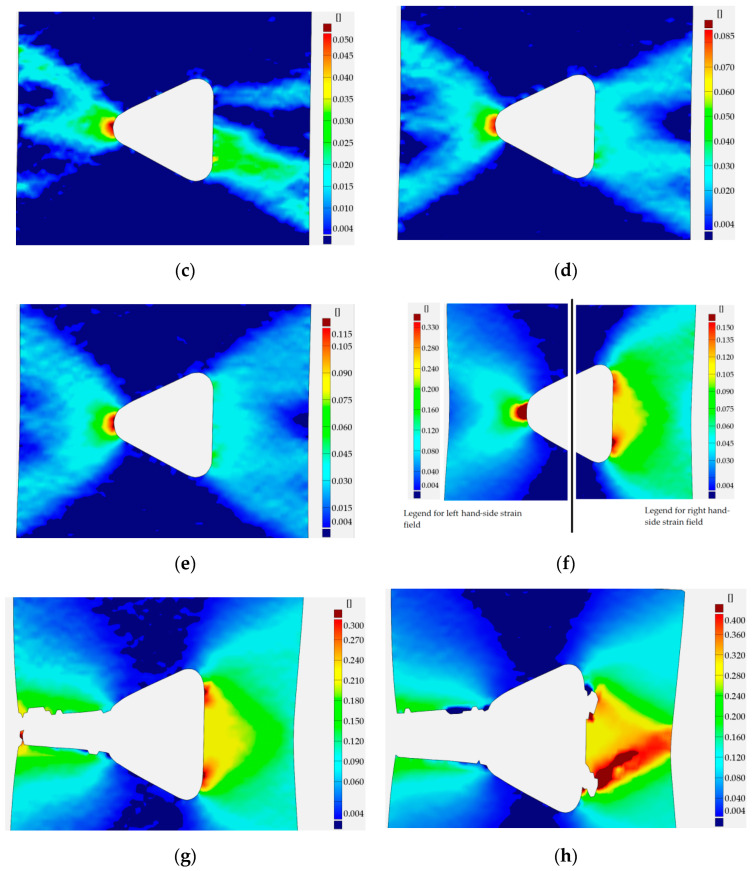
Strains field on investigated surface of plate made of S235JR + N with triangular hole calculated with the use of DIC; total vertical displacement u_MTS_ and corresponding vertical ε_TOT_ (in brackets): (**a**) 0.83 (0.46); (**b**) 0.91 (0.505); (**c**) 1.16 (0.644); (**d**) 1.58 (0.876); (**e**) 2.08 (1.16); (**f**) 5.91 (3.28); (**g**) 10.43 (5.79); and (**h**) 15.1 (8.39).

Comparing the strains fields (Figure 25) with the σ–ε curve (Figure 14), it can also be observed that plastification of the weakened transversal cross-section of the plate was achieved between Points 3 (Figure 25a) and 4 (Figure 25b). Further increase of the load leads to a similar growth of plastic zones, as described for the previous examples with circular and rectangular holes. Higher values of strains occur on the V1 corner side. It leads to crack initiation in point V1 for total strain ε_TOT_ = 3.28 (Point 5 in Figure 14a, Figure 15a and Figure 25f). Distribution of the strains directly after the failure of the left side of the sample is given in Figure 25g. This image corresponds to Point 6 of the σ–ε curve (Figure 14 and Figure 15b). The strain field at the moment of the crack propagation (after exceeding Point 7; Figure 14a) as well as the visible crack (dark red) and further crack path (red) is given in Figure 25h.

**Figure 26:**
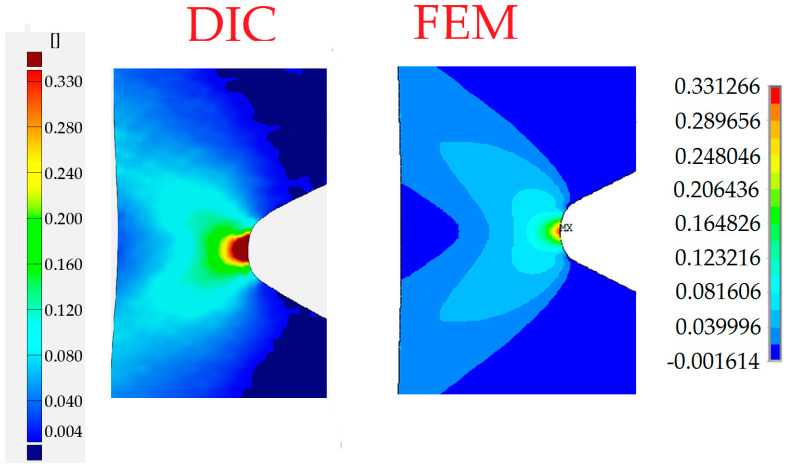
Comparison of DIC–GOM and finite element analysis results.

In the case of the sample with a triangular hole, the finite element model of the whole sample was analyzed. The comparison of the total vertical strains is presented in Figure 26. For the chosen value of the total vertical strains, the good agreement of contour lines was also observed.

In all presented examples, the total vertical strains were compared—these were of the highest value due to the applied tension, also in the vertical direction. In the case of holes of circular and square shapes, almost symmetric—with respect to the vertical axis—results were observed in the experiment and obtained in numerical analysis. The comparison exhibits the fairly good agreement of the experimental and numerical results. In the case of the triangular void, the observed growth of plastic zones is a kind of mixture of the behavior of the tensed sample with circular hole (Corner V1) and the sample with the square hole (Corners V2 and V3). Here, fairly good agreement of the result was also spotted.

### 6.2. DIC Analyses of S355MC and 41Cr4 Steels

**Figure 27:**
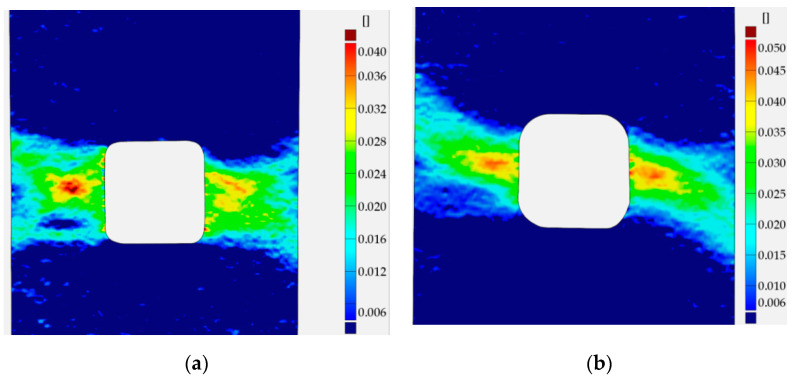
Distribution of strain field (DIC) in samples with square holes made of S355MC steel: (**a**) Sample 9 with corner fillet radius R2; and (**b**) Sample 10 with corner fillet radius R4.

**Figure 28:**
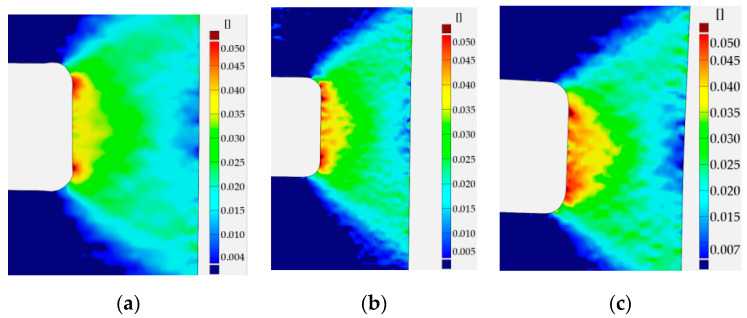
Distribution of strain field (DIC) in samples with square holes made of 41Cr4 steels: (**a**) 41Cr4 + AC, Sample 11; (**b**) 41Cr4 + AC, Sample 12; and (**c**) 41Cr4 + QT, Sample 14.

Similar analyses were performed for specimens with square holes made of S355MC and 41Cr4 steels. In these investigated examples, the different development of the plastic zones was observed for different materials. These distributions for the range of the strain hardening are presented in Figure 27a for S355MC with corner radius R2, Figure 27b for S355MC with corner radius R4, Figure 28a,b for 41Cr4 + AC, and Figure 28c for 41Cr4 + QT.

**Figure 29:**
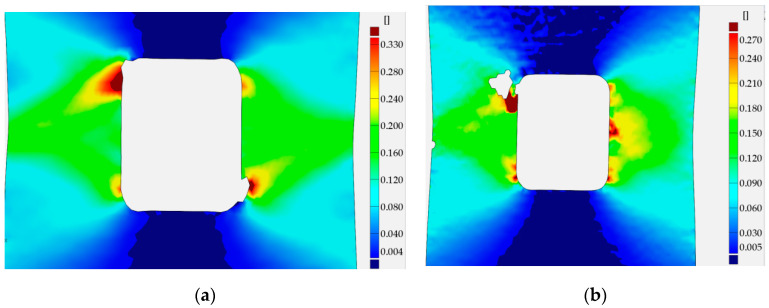
Distribution of strain field (DIC) in samples with square holes made of 41Cr4 + AC steel: (**a**) Sample 11; and (**b**) Sample 12.

**Figure 30:**
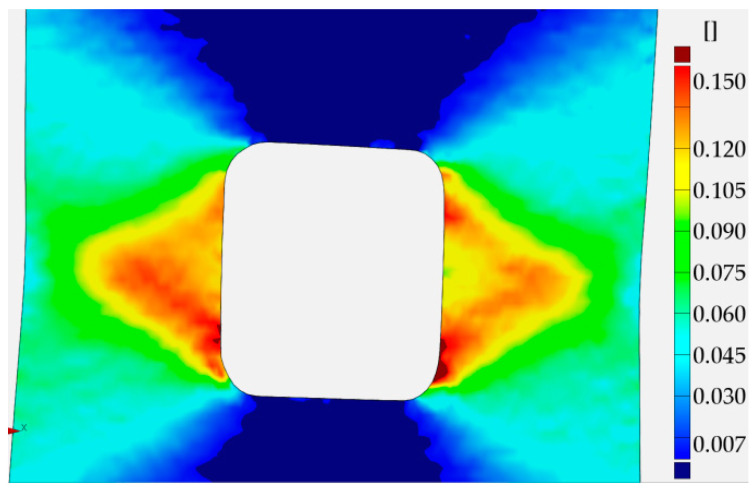
Distribution of strain field (DIC) in samples with square holes made of 41Cr4 + QT steel.

In the case of the high-strength steel 41Cr4, high strain concentrations occurred in the notches (Figure 28), similar to was observed for S235JR + N (Figure 23). In addition, similar sample behavior during the phase of crack nucleation was observed for both materials (Figure 29, 41Cr4 + AC; Figure 30, 41Cr4 + QT; Figure 23g,h, S235JR + N). The first cracks in these three examples nucleated at the notch and propagated along the lines inclined with approximately 31° (Figure 13c; S235JR + N) and 26° (Figure 19a, 41Cr4 + AC; Figure 19e, 41Cr4 + QT). In the remaining two samples made of 41Cr4 + AC, the first cracks had more complex shapes (Figure 19b,c). In the DIC analyses, it was observed that laser cutting had a significant influence on the behavior of the softened 41Cr4 steel. The locally concentrated high temperature during laser cutting caused the hardening of the material around the hole. More vulnerable for such manufacturing was softened 41Cr4 + AC steel due to the large difference in hardness of the sample. It resulted in occurring local concentration of strains through the whole vertical edges of the square holes. However, it should be noted that, before primary crack initiation, the highest stress concentration was observed in the surroundings of the ends of the corner fillet radii (Figure 28). The initiation of the primary cracks (Figure 29; areas in which strains exceed 0.3 and white areas in which results from DIC are missing designate damaged zones of the samples) resulted in the redistribution of the stresses and caused in two samples (Samples 12 and 13) appearance of stress concentration in the new point in the middle of the vertical edge of the hole (Figure 29b). It should be also noted that, at this point, laser cutting was started, and, finally, the second crack was initiated at this point. These behaviors of the 41Cr4 + AC and 41Cr4 + QT steels can be attributed to the relatively high sensitivity of the presence of the notches caused by material hardening.

The significant differences in the plastic strain distributions in comparison with other investigated samples were observed for samples made of S355MC (Figure 27). In these cases, the strain concentrations determined by GOM were not at the notches but in the mid-part of the weakened cross-section far from the edge of the square hole. Consequently, the final cracks occurred in the middle of the weakened cross-sections (Figure 17b). The change of the notch radius (from R2 to R4) results in a slight difference in the plastic strain growth. These effects confirmed the low sensitivity of S355MC on the notch presence and laser cutting.

## 7. Microstructure and Fracture Analysis

**Figure 31:**
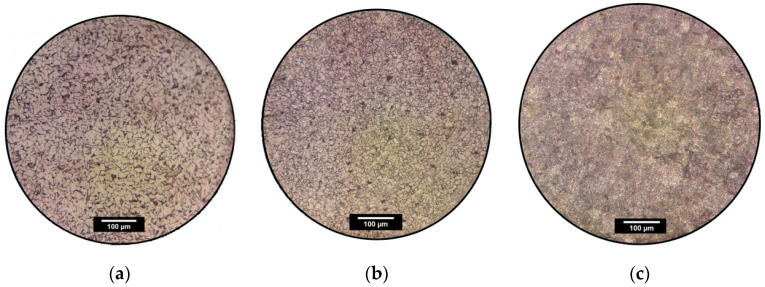
Microstructures of selected steels to experimental tests (250× magnification): (**a**) S235JR + N; (**b**) S355MC; and (**c**) 41Cr4.

Materials used in the experimental tests belong to different groups of steels. Therefore, an analysis of the microstructure of materials in the delivery condition was prepared. In the first step, the samples of material were cut out by mechanical cutting without providing heat, which saved the material from structural changes. In the next step, the samples were embedded in the duracryl polymer. The surfaces of metals were grinded by the sandpaper with gradation varying from 120 to 2500. After that, the polishing process was performed with the use of aluminum oxide (alumina). To expose microstructure features such as the size of grains and phases of material, 5% of the Nital solution was used, which is the typical reagent applied for revealing the microstructure of carbon steels. Microstructure photos were taken by metallurgical microscope MET-3 PZO with additional camera grip, and the results are presented in Figure 31.

The microstructures presented in Figure 1 shows the influence of the chemical composition of the steel on the grain sizes. The observed structural components are grains of ferrite (light gray) and perlite (black). For common structural steels S235JR + N (Figure 31a), S355MC (Figure 31b), and 41Cr4 (Figure 31c) it is possible to notice the large grains. For S355MC, it is associated with the presence of vanadium (V), niobium (Nb), and titanium (Ti) as additive components causing the grain refinement. In the case of 41Cr4 steel, the main alloying additive is chromium (1.04%). Due to the higher content and stronger influence, the microstructure is more fine-grained than in other cases of steels.

**Figure 32:**
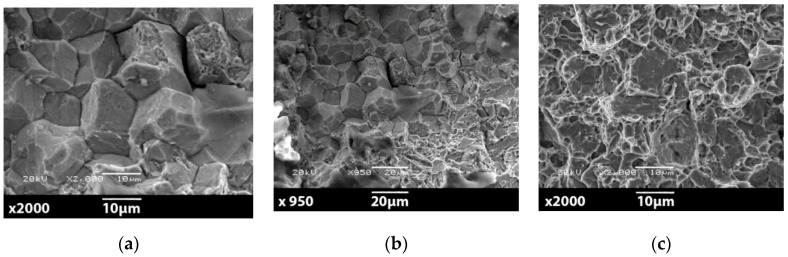
Fracture of 41Cr4 + Q—54 HRC: (**a**) on the notch-edge; (**b**) transition between two types of crack; and (**c**) central area of sample.

**Figure 33:**
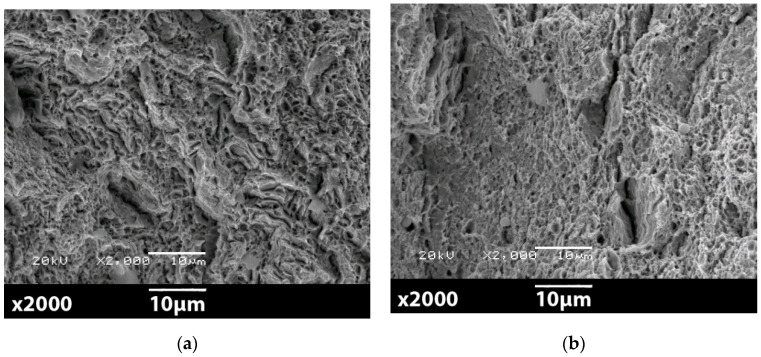
Fracture of 41Cr4 (HT), 35 HRC: (**a**) on the notch-edge; and (**b**) in central area of sample.

The surfaces of ruptures were observed for samples after heat treatment process, namely for 41Cr4 + Q and 41Cr4 + QT. The study was made with the use of the scanning electron microscope (SEM) JOEL JSM5510LV. The results for 41Cr4 + Q are presented in Figure 32 and for the 41Cr4 + QT in Figure 33. The sample 41Cr4 + Q had typical martensite microstructure and 41Cr4 + QT had tempered martensite microstructure.

Figure 32 presents the microscopic view of brittle fracture of the sample 41Cr4 + Q with distinctive two characteristic zones. The zone in Figure 32a presents intercrystalline rupture on the edge of the sample in the notch area and goes to boundary rupture in Figure 32b. In Figure 32b, it is possible to identify on boundary two types of crack—intercrystalline and quasi-cleavage fracture surface. In Figure 32c, the typical bright, reflective facets are spotted, which are associated with low-energy brittle fracture. To a large extent, the surface of the broken sample represents quasi-cleavage fracture; only in the notch-edge, the small area of the intercrystalline fracture is present. The process of the sample failure occurred suddenly with only little resilient strain and without local reduction of cross-section (Figure 19f).

The result of the 41Cr4 + QT sample fracture (see Figure 33) investigation is a ductile fracture in the entire surface. This type of rupture is determined by the presence of fibrous fracture with visible micro-voids. Together with the growth of applied tensile stress, microvoids enlarged and in some part coalescence in the form of strip/band appeared. The coalescence, seen as ductile dimples, was observed in the rupture, which is the indication of the presence of ductile overload of the material. This demonstrates the very good plasticity of the investigated material and the high ability to deform. As a result, the investigated sample absorbed the deformation energy and showed pronounced plastic deformation with the necking effect before the failure. In Figure 19e, visible reduction of the cross-section after failure is presented.

## 8. Discussion

The presented study is focused on the two problems—determination and influence of the SCFs and notch sensitivity on the material behavior, plastic deformation growth, and failure mode, and possibilities of applying DIC in the experimental analyses of the recent materials. The presented analytical and numerical FE calculations demonstrate that there are still unsolved issues related to the stress concentrators with typical and common shapes of notches. The application of the finite-width correction factors also may not guarantee an accurate assessment of SCF. This can be seen on the example of a plate with a square hole in which the application of the theoretical formulas and the correction factor leads to the non-conservative estimation of the SCF (Figure 5a and Table 5). On the other hand, the use of the theoretical model for infinite plate leads to a large overestimation of the SCF, which may lead to the overestimation of the dimensions of the designed element. In such a situation, in the design process of structures, particularly subjected to fatigue loadings, the application of the FEM analysis seems to be the most reasonable approach. It was also observed that, in the case of the holes in which the edges are parallel to the tension, the direction of the major stress in the zone of the highest stresses is rotated with respect to the tension direction. In such a situation (rectangular and triangular holes), the SCF calculated from the principal stresses gives higher values than the SCF calculated form the vertical tensile stress.

In the investigated samples made of S235JR + N, the SCF *K_t_* for the square hole (*K_t_* = 2.46) was larger from the SCF for the circular hole (*K_t_* = 2.34—see Table 5). However, it should be noted that the fillet radius R5 of square corners is not optimal for the chosen sample width and the hole size (see Figure 5a). The optimal radius for such geometry appears for the ratio *R*/*b* = 0.4, which corresponds to fillet radius R12. In the case of the optimal square hole, with fillet radius R12, the SCF is equal to *K_t_* = 2.17 (Figure 5a) and is smaller than for equivalent sample with the circular hole with a diameter equal to the distance opposite straight edges of the square. Moreover, the use of the square hole results also in a smaller weight of the sample. It demonstrates that the use of square holes with fillet corners in the optimization of the fatigue strength and weight of a structure gives clear benefits in comparison with circular holes. Rectangular holes seem to be even more advantageous than square holes, with a longer side in the tension direction or oval holes [21].

The tensile tests performed for plates with holes demonstrated the influence of the stress concentrators. In the first part of the σ–ε curves for the samples with holes made of S235JR + N, two linear parts with different stiffness can be observed. In the case of the plate with the circular hole, the curve bends at the value of σ_notch,MAX_ = σ_notch,NOM_·*K_t_* (*K_t_* was determined by FEM) close to the 300 MPa (Figure 10b). In the case of the plate with the square hole, such stress was lower than 300 MPa (Figure 11b), but it was caused by slight non-symmetrical tension of the plate (loosing of the mounting screws in the grip). This effect occurred in the first part of the test and has been observed after the test on the displacement and strain maps obtained from DIC analysis (see Figure 23a,b). Increased load on the left-hand side of the sample caused higher stresses on the left side of the hole. The effect of stress concentration was also studied at the example of the triangular hole. In this case, two points were examined—Corners V1 and V3. In the case of the V1 corner, the σ_notch,MAX_ was again very close (about 295 MPa—Figure 14b) to the value of the Yield plateau. A similar situation was in Corner V3, which has similar shape and effect as the edge of the square hole, in which σ_notch,MAX_ ≈ 289 MPa. This value is lower than 300 MPa because plasticity in the V3 corner begins when strain hardening occurs in the surrounding of the V1 corner (Figure 25). The analysis of the obtained σ–ε curves for the sample without and with a stress concentrator with the use of SCF value calculated by FEM analysis justifies that estimated SCF agrees with the results achieved from the experiments. Similar numerical and experimental tests were performed for S355MC and 41Cr4 steels. However, in both cases, the σ–ε curves did not show such significant changes in the σ–ε shape caused by the stress concentrations. Because of this, it was difficult to achieve satisfactory accuracy of total stress (strain) determination at which plastification around the notches occurs.

The performed experimental tests were made with the simultaneous use of the DIC technique. Recently, there are available professional 3D DIC systems with fast release and high-quality cameras. However, the main aim of the study was to evaluate the possibilities of a simple DIC system based on the common Nikon D90 camera. Such instrumentation has an advantage over other professional SHM devices due to the accessibility and price. In comparison with the professional DIC systems, the largest disadvantage of the used hardware is its sensitiveness (camera picture resolution, etc.) and applicability only to the flat surfaces. Due to the resolution of the Nikon D90 camera, the DIC analyses were possible only after the occurrence of the considerable plastic deformations. Because of this, such system is practically not suitable to the hardened high-strength steels (such as 41Cr4 + Q) in which plastic strains practically did not occur. On the other hand, in the case of heat-treated (+QT) and softened (+AC) high-strength 41Cr4 steel, it is possible to determine the displacement and strain fields of the investigated surfaces with satisfactory accuracy. It should be also noted, that accuracy of the results for samples made of 41Cr4 was reduced due to the images being taken through the closed cover of the testing machine (safety requirements). In the case of the S355MC steel, the limit for DIC application was the chipping resistance of the paint to peel off from the tested surface. The microalloyed fine-grain structural S355MC steel exhibit significant thinning in the volumes weakened by hole, which can be attributed to its high ductility. This leads to the faster degradation of the varnish layer much earlier than the crack nucleation. From this moment, the DIC analyses calculate displacements related not only to the sample but also to the damaged varnish layer. Generally, in such zones, in which the varnish layer is peeled off from the surface the DIC analysis will reveal large deformations or failure. It remains in disagreement with the experiment and caused by the fact that the strain field calculation in the DIC method is based on the deformation of the surface coating. This effect also occurred in the final part of the test of the plate with a square hole made of S235JR + N. For the two remaining samples made of S235JR + N (circular and triangular holes), the DIC analyses were possible almost to the final failure of the samples. In these cases, it allows for correct detection and tracking crack initiation and growth.

Except for the sample made of 41Cr4 + Q, the DIC method allows for the fairly good determination of plastic zone growth, the location of zones where the failure may occur, and in some cases, it even allows for the prediction of the final failure form. The obtained distributions of plastic zones were compared with the results of the FEM analyses. The largest difficulties with the FEM analysis were related to the modeling of the material behavior and the elaboration of the equivalent model in the range of the large plastic deformations. In addition, it should be noted that the simple, well-established material models are only applicable to a limited strain range [40]. To obtain convergence of the FEM solution, the simplification described in Section 5 was made in the material model assumption. These simplifications result in some differences (i.e., total displacement) with experiments but the shape of the plastic zones under particular total strain around the notch was in good agreement with the DIC analyses.

The performed experimental tensile tests, microstructure analyses, and DIC studies revealed the disadvantageous influence of martensite structure after hardening on the structure strength and notch sensitivity. Such martensite structure, formed during the hardening process of the material, is very hard, by what is exposed for low-energy brittle fractures. However, it was also observed that martensite transformation may occur during a laser cutting of the hardenable steel (such as 41Cr4) and may significantly decrease their mechanical properties due to the increased sensitivity of the material for the notches. In the case of such material, it is important to start the laser cutting in an area where no stress concentration may appear It was also observed that the intensity of the laser cutting on notch sensitivity was much stronger in the softened 41Cr4 + AC steel than heat-treated 41Cr4 + QT one. On the other hand, there are also materials (i.e., S355MC) that are insensitive for laser cutting. The remarks given in this paragraph are fully confirmed by the microstructure and fracture studies given in Section 7, revealing the presence of the respective mild or hard particles.

## 9. Conclusions

The performed study was focused on the possibilities of application and the reliability of the DIC system for the investigations of different steels—mild S235JR+N steel, microalloyed fine-grain S355MC, and different heat-treated high-strength 41Cr4 steel subjected to tension. With the use of the DIC system, the influence of the notch presence and its geometry were studied. The studies were performed for thin flat samples with circular, square, and triangular openings. The following conclusions can be drawn:

(1) The application of DIC with a common digital camera can be effectively applied for evolution of plastic zones and damage detection for mild, as well as normalized and quench and tempered in higher temperatures high-strength steels.
(2) The effectiveness of the DIC usage increases with the decrease of the hardness (increase of the minimal elongation) of the material.
(3) In the case of the quenched steels, the resolution of the common camera seems to be too low, or the test should be made with much lower velocity of the tension.
(4) The general forms of the plastic zone obtained from DIC were in good agreement with FE simulations.
(5) DIC can be effectively used as an additional control measurement tool for experimental tests.
(6) The disadvantage of DIC analyses is that the deformation of varnish layer is measured, if varnish layer peels off from the surface the method may lead to the misleading results.
(7) It is possible to predict the dangerous zones in the structure, and it is often possible to predict the final fracture form before the crack would nucleate with the use of the DIC system as well.
(8) The performed analytical and FE calculation revealed that theoretical models for calculations of the influence of typical notches may result in not proper values for SCF.
(9) Microalloyed fine grain S355MC steel revealed very low sensitivity for notch presence.
(10) Steel S355MC revealed no sensitivity for laser cutting.
(11) Steel 41Cr4 revealed the strong local influence of the laser cutting on the microstructure (can cause local hardening in HAZ, particularly in starting point of cutting), and laser significantly increases notch sensitivity of 41Cr4 when it is in softened state.

## Figures and Tables

**Table 1:** Chemical composition of investigated steels.

Chemical Components of Steel [%]													
Material	C	Mn	Si	P	S	Cu	Al	Cr	Ni	Sn	Nb	Ti	Fe
S235JR + N	0.12	0.52	0.009	0.012	0.011	0.05	0.047	0.02	0.013	0.0036	-	0.001	residue
S355MC	0.07	0.84	0.017	0.013	0.007	0.068	0.072	0.057	0.033	0.005	0.032	0.001	residue
41Cr4	0.39	0.673	0.242	0.009	0.01	0.281	0.035	1.04	-	0.013	-	-	residue

**Table 2:** Mechanical properties of investigated steels [79,80,81].

Material		Upper Yield Limit *Y_eH_* [MPa]	Tensile Strength *R_m_* [MPa]	*Y_eH_*/*R_m_* [-]	Elongation *A* [%]	*HB*^1^ [-]
EN	AISI, ASTM					
S235JR + N	A283 Gr.C	338	433	0.78	31.8	Max. 150
S355MC	Gr.50/050XLK	355	430	0.83	Min. 23%	Max. 187
41Cr4 + AC	5140	496	619	0.80	25	Max. 240

^1^ HB, Brinell Hardness.

**Table 3:** Description of investigated samples.

No. of Sample	Geometry	Hole Type	Material	Loading Rate [mm/min]	Remarks
1–3	Figure 1a	Not applicable ^1^	S235JR + N	1.0	-
4	Figure 1b	Circular φ30 mm	S235JR + N	1.0	-
5	Figure 1b	Square 30 × 30 mm	S235JR + N	1.0	Corner fillet radii 5 mm
6	Figure 1b	Equilateral Triangular—span length 30 mm	S235JR + N	1.0	Corner fillet radii 5 mm
7	Figure 2a	Not applicable ^1^	S355MC	1.0	Cut perpendicular to the rolling direction
8	Figure 2a	Not applicable ^1^	S355MC	1.0	Cut along the rolling direction
9	Figure 2b	Square 15 × 15 mm	S355MC	0.5	Corner fillet radii 2 mm
10	Figure 2b	Square 15 × 15 mm	S355MC	0.5	Corner fillet radii 4 mm
11–13	Figure 2c	Square 15 × 15 mm	41Cr4+AC	0.5	Corner fillet radii 2 mm
14	Figure 2c	Square 15 × 15 mm	41Cr4+QT	0.5	Corner fillet radii 2 mm
15	Figure 2c	Square 15 × 15 mm	41Cr4+Q	0.5	Corner fillet radii 2 mm

^1^ Geometry following the standard [82]; sample without hole.

**Table 4:** Finite-width correction factors for mathematical model given in Equations (3) and (4).

0.5*a*/*W*	*C_f_*
0.05	1.01
0.1	1.03
0.15	1.05
0.2	1.09
0.25	1.13

**Table 5:** Stress concentration factor values for tested specimens.

No. of Sample	Material	Notch Geometry and Fillet Radius, in [mm]	Notch Sensitivity of Material	$K_{t}^{\mathrm{INF}}$ Infinity Panel ^1^	$K_{t}$ Finite-Width Panel ^2^	$K_{t}^{\sigma \;Y}$ SCF FEM ^3^	$K_{t}^{\sigma \;1}$ SCF FEM ^4^
4	S235JR + N	Circular φ30 mm	Moderate	3.00	2.31	2.34	2.34
5	S235JR + N	Square 30 × 30, R5	Moderate	3.28	2.33	2.37	2.46
6	S235JR + N	Triangular, R5	Moderate	-	-	V1: 3.58 V3: 2.30	V1: 3.58 V3: 2.37
9	S355MC	Square 15 × 15, R2	Low	3.50	2.48	2.51	2.59
10	S355MC	Square 15 × 15, R4	Low	2.94	2.08	2.20	2.25
11–13	41Cr4 (N)	Square 15 × 15, R2	High	3.50	2.48	2.50	2.59
14	41Cr4 (T)	Square 15 × 15, R2	Very High	3.50	2.48	2.50	2.59
15	41Cr4 (H)	Square 15 × 15, R2	Extremal	3.50	2.48	2.50	2.59

^1^ SCF was calculated with the use of the analytical model for plate with infinite width; ^2^ SCF was calculated with the use of the analytical model for plate with finite width; ^3^ SCF was calculated with the use of FEM and vertical stress σY; and ^4^ SCF was calculated with the use of FEM and the major stress σ1. The definitions of V1 and V3 corners are given in Figure 6c,d.
